# Coupled Vacancy and Phonon‐Scattering Engineering Drive Defect Evolution Toward Multifunctional High‐Performance Bi_2_Te_3_ Thermoelectrics

**DOI:** 10.1002/advs.75529

**Published:** 2026-05-04

**Authors:** Ruiheng Li, Minwen Yang, Huangshui Ma, Jie Zheng, Shengqiang Cui, Xiaobo Tan, Xuri Rao, Xiang An, Zongxiang Kan, Siqi Huo, Jing Shuai, Min Hong, Ran Ang

**Affiliations:** ^1^ Key Laboratory of Radiation Physics and Technology Ministry of Education Institute of Nuclear Science and Technology Sichuan University Chengdu China; ^2^ School of Materials Sun Yat‐Sen University Shenzhen China; ^3^ Centre for Future Materials School of Science Engineering and Digital Technologies University of Southern Queensland Springfield Campus Queensland Australia; ^4^ Xianghe Environmental Protection Industrial Park Xianghe China; ^5^ Institute of New Energy and Low‐Carbon Technology Sichuan University Chengdu China; ^6^ College of Physics Sichuan University Chengdu China

**Keywords:** Bi_2_Te_3_‐based materials, multifunctional thermoelectric devices, phonon scattering, swapped bilayer structures, thermoelectrics, vacancy compensation

## Abstract

Thermoelectric (TE) materials with high‐efficiency solid‐state cooling and low‐grade heat harvesting are crucial for sustainable energy technologies. Although Bi_2_Te_3_‐based compounds remain the only commercially viable near‐room‐temperature TE system, their deployment is still constrained by moderate conversion efficiency, limited mechanical robustness, and restricted multifunctionality. Here, we propose a dual‐regulation strategy that integrates intermetallic ZnSb and Se dopants to synergistically modulate carrier and phonon transport in Bi_0.4_Sb_1.6_Te_3.01_. ZnSb incorporation compensates for Sb vacancies, suppresses Te volatilization, and decreases carrier concentration, thereby enhancing the Seebeck coefficient and power factor. Concurrently, Se doping introduces hierarchical phonon‐scattering centers and induces swapped‐bilayer configurations near twin boundaries, strengthening interlayer coupling and improving mechanical integrity. The optimized Bi_0.4_Sb_1.6_Te_2.97_Se_0.04_ + 0.15% ZnSb achieves a peak *zT* of ∼1.51 at 353 K and an average *zT* of ∼1.47 below 403 K, together with high Vickers hardness (∼97 Hv) and compressive strength (∼188 MPa). A finite‐element‐optimized multifunctional device further delivers a maximum cooling temperature difference of ∼70 K at 303 K and a power‐generation efficiency of ∼7.1% under a 208 K temperature gradient, with exceptional stability under room‐temperature wearable conditions. This study establishes a scalable design framework linking atomic‐scale defect manipulation to device‐level performance for practical, multifunctional Bi_2_Te_3_‐based thermoelectrics.

## Graphical abstract

ZnSb reduces carrier concentration and suppresses Te volatilization, while Se substitution within QLs softens phonons and enhances anharmonicity. Swapped bilayers near twin boundaries strengthen phonon scattering and interlayer bonding. As a result, the optimal sample achieves an ultra‐high *zT* ∼1.51, cooling temperature difference of ∼70 K, power‐generation efficiency of ∼7.1%, and robust room‐temperature power output.

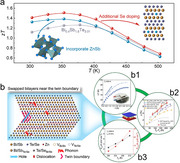

## 1 Introduction

In response to the dual challenges of energy utilization and thermal management, developing materials and devices capable of efficient waste heat recovery and precise temperature control is of paramount importance [1, 2]. Thermoelectric (TE) materials, which can directly convert heat into electricity, inherently offer dual functionalities of power generation and solid‐state cooling, providing a unique avenue for integrated energy conversion and temperature regulation [3, 4, 5, 6]. The performance of TE materials is primarily characterized by the dimensionless figure of merit, *zT* = *σS* ^2^ *T*/*κ*, where *σ*, *S*, *T*, and *κ* denote the electrical conductivity, Seebeck coefficient, absolute temperature, and total thermal conductivity, respectively (*κ = κ*_e_ *+ κ*_l_ *+ κ*_b_, with *κ*_e_, *κ*_l_, and *κ*_b_ representing the electronic, lattice, and bipolar contributions) [7, 8]. Maximizing *zT* requires a simultaneous enhancement of the power factor (*PF = σS* ^2^) and suppression of *κ*, which directly translates into improved device efficiency in both power generation and cooling applications [9].

At near‐room temperature, Bi_2_Te_3_ and its alloys remain the most promising TE systems for practical applications [10]. They have long underpinned the industrialization of solid‐state cooling and exhibit significant potential for low‐grade waste heat recovery and portable energy conversion [5]. Although lightweight and cost‐effective Mg_3_(Bi, Sb)_2_ compounds demonstrate comparable TE performance, their long‐term stability and device reliability remain unproven [11, 12]. Despite extensive optimization, a substantial performance gap between commercial Bi_2_Te_3_ materials and their laboratory‐scale counterparts, particularly for p‐type compositions, whose *PF* and overall *zT* values remain far below research‐grade samples [13, 14]. Furthermore, commercial Bi_2_Te_3_ typically features coarse grains and strong texture, which collectively compromise mechanical integrity [15]. Such microstructural limitations not only undermine the long‐term reliability of solid‐state cooling devices but also restrict stable power output in energy‐harvesting applications [16]. With the rapid expansion of Bi_2_Te_3_‐based TE materials into emerging fields—including 5G optical communication modules, nuclear waste heat recovery, and flexible wearable electronics—the development of multifunctional TE materials and devices that simultaneously deliver high energy efficiency, mechanical robustness, and environmental adaptability has become critical for next‐generation TE technologies [17, 18, 19, 20, 21, 22, 23, 24].

Previous studies have shown that doping p‐type Bi_2_Te_3_ with a slight excess of Te (∼1 mol%, e.g., Bi_0.4_Sb_1.6_Te_3.01_) induces a self‐compensation effect, mitigating the “donor‐like” behavior introduced during high‐energy ball milling. This strategy effectively optimizes the hole concentration (*p*_H_) and enhances the *PF*. Additionally, during powder sintering, volatilized Te can induce microporous and dislocation arrays, which scatter mid‐frequency phonons and reduce *κ*_l_ [25, 26]. Despite these advantages, the Te self‐compensation approach has significant limitations. Owing to Te's high saturated vapor pressure, substantial composition fluctuations and processing instabilities often occur during sintering. Te segregation at grain boundaries promotes grain‐boundary sliding, while associated voids and microcracks degrade both Hall mobility (*µ*_H_) and mechanical integrity [27]. Therefore, relying solely on the self‐compensation—analogous to the Sn self‐compensation mechanism in SnTe—offers limited potential for achieving both high TE performance and structural stability [28]. These challenges are especially critical for TE devices requiring long‐term stability and high output in cooling and power generation applications. To address this, researchers have introduced low‐*κ* secondary phases or exogenous dopants (e.g., ZIF‐8, Cu_3_SbSe_3_, NaBiS_2_, Ag_5_Te_3,_ and S/Se) to simultaneously enhance electrical performance and mechanical robustness through solid‐solution strengthening and defect engineering [25, 29, 30, 31, 32, 33].

**FIGURE 1:**
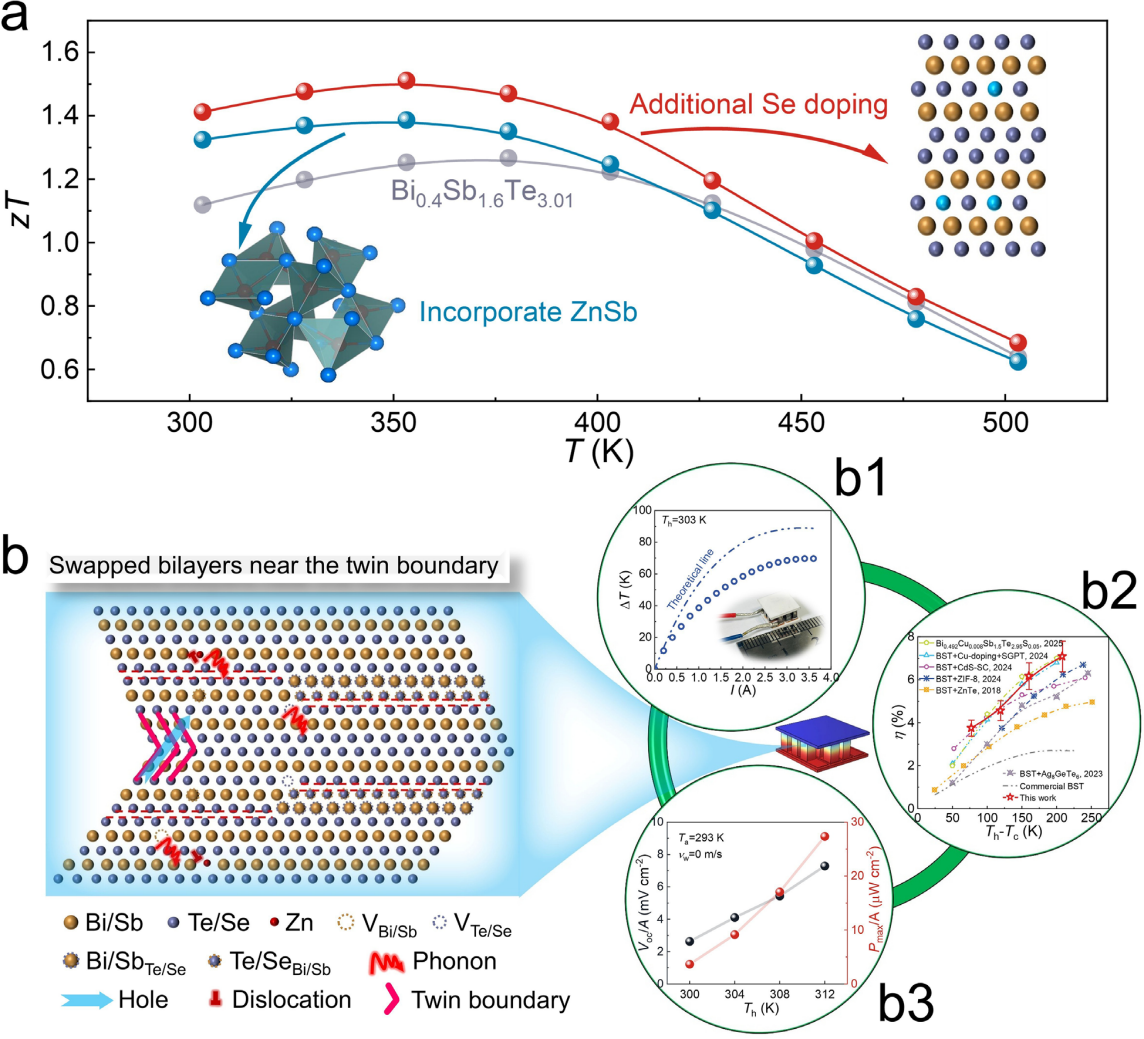
Thermoelectric performance of Bi_0.4_Sb_1.6_Te_3.01‐_ *_y_* Se*_y_* + *x*% ZnSb materials and mechanism diagram. (a) Temperature‐dependent *zT* values of pristine Bi_0.4_Sb_1.6_Te_3.01_ and optimally engineered Bi_2_Te_3_‐based alloys. (b) Schematic illustration of the coupled hole‐phonon scattering processes in the Bi_0.4_Sb_1.6_Te_2.97_Se_0.04_ + 0.15% ZnSb sample, highlighting the formation of swapped bilayers (SBs) near the twin boundary (TB) and device‐level performance optimization enabled by finite element simulations. (b1) Comparison between the simulated (dashed line) and experimentally measured cooling temperature difference (Δ*T*) as a function of current (*I*) at a fixed hot‐side temperature (*T*_h_) of 303 K. (b2) Measured TE conversion efficiency (*η*) of the Bi_0.4_Sb_1.6_Te_2.97_Se_0.04_ + 0.15% ZnSb/Bi_2_Te_2.7_Se_0.3_ device at different temperature gradients *T*_h_‐*T*_c_ (*T*_c_ is the cold‐side temperature), benchmarked against representative literature reports. Abbreviations: BST, bismuth antimony telluride; SC, super‐tetrahedron cluster; SGPT, Sn_1/3_Ge_1/3_Pb_1/3_Te; ZIF, zeolitic imidazolate framework [29, 32, 33, 36, 37, 38, 39]. (b3) Voltage density (*V*_oc_/*A*) and maximum power density (*P*_max_/*A*) as a function of *T*_h_ for the Bi_0.4_Sb_1.6_Te_2.97_Se_0.04_ + 0.15% ZnSb/Bi_2_Te_2.7_Se_0.3_ device under windless indoor conditions (*v*_w_ = 0 m/s) without additional cold‐side cooling. Here, *A*, *V*_oc_, and *P*_max_ denote the cross‐sectional area of the device, open‐circuit voltage, and maximum output power, respectively. The ambient temperature (*T*_a_) is 293 K.

In this study, the binary intermetallic compound ZnSb, featuring a simple orthorhombic crystal structure (space group *Pbca*), is employed as a functional tuning unit [34]. As a classic Zn‐Sb‐based TE compound, ZnSb exhibits pronounced lattice anharmonicity, intrinsically suppressing *κ*_l_ [35]. Compared with complex multicomponent systems prone to phase separation or heterogeneous phase formation, the binary chemistry of ZnSb enables continuous solid solution or stable recombination with the Bi‐Sb‐Te matrix, providing enhanced synthetic controllability. Experimentally, ZnSb incorporation reduces *p*_H_ via Zn substitution at Sb sites, Sb vacancy filling, and antisite defect formation (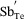). Among these, Sb vacancy filling plays the dominant role, enhancing carrier scattering while simultaneously suppressing Te volatilization and micropore formation, thereby increasing *S* and improving *PF*. Further introduction of Se modulates lattice dynamics and intensifies phonon scattering. Phonon dispersion calculations indicate that Se substitutes Te within the quintuple layers (QLs), rather than at the van der Waals (vdW) gaps, softens acoustic branches and enhances lattice anharmonicity, achieving maximal *κ*_l_ suppression. In combination with multiple scattering sources—including grain boundaries, high‐density dislocations, stacking faults (SFs), and low defect formation energies (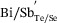)—this facilitates swapped‐bilayer (SB) formation near twin boundaries (TBs), which strengthens phonon scattering without compromising *µ*_H_ and reinforces interlayer coupling, enhancing mechanical robustness. Benefiting from the synergistic ZnSb–Se regulation, the optimized Bi_0.4_Sb_1.6_Te_2.97_Se_0.04_ + 0.15% ZnSb exhibits a peak *zT* of ∼1.51 (average *zT*_ave_ ∼1.47 below 403 K), high Vickers hardness (∼97 Hv), and compressive strength (∼188 MPa) (Figure 1a). A multifunctional TE device fabricated from this material achieves a maximum temperature difference (Δ*T*_max_) of ∼70 K at a hot‐side temperature (*T*_h_) of 303 K and a conversion efficiency (*η*) of ∼7.1% under a 208 K temperature gradient, maintaining stable power output at room temperature with a power density of 27.3 µW cm^−2^ at *T*_h_ = 312 K (Figure 1b). This work establishes a concise, scalable dual‐regulation strategy linking atomic‐scale defect engineering with device‐level optimization, providing a practical pathway for high‐performance, mechanically robust, multifunctional TE materials operating near room temperature.

## 2 Results and Discussion

As shown in Figure S1, the X‐ray diffraction (XRD) patterns of Bi_0.4_Sb_1.6_Te_3.01‐_ *_y_* Se*_y_* + *x*% ZnSb indicate that all samples can be well indexed to the rhombohedral Bi‐Sb‐Te phase (PDF#49‐1713, space group $R\bar{3}m$) [9]. Within the detection limits of XRD, all doped samples retain a single‐phase structure without detectable secondary phases. A closer inspection of the diffraction peak near 28° reveals a gradual shift toward higher angles with increasing ZnSb content, which is attributed to the incorporation of a smaller Zn atom (atomic radius ∼125 pm) into the lattice, as will be elaborated below. Interestingly, at low Se doping levels, the main diffraction peak initially shifts to lower angles and subsequently moves to higher angles as the Se content increases. This non‐monotonic behavior suggests that at low Se concentrations, the relatively high volatility of Se during synthesis introduces local lattice distortion and alters defect configurations, possibly increasing the concentration of 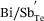 antisite defects, which can lead to lattice expansion. At higher Se doping levels, Se preferentially substitutes for Te, and the smaller atomic radius of Se dominates, resulting in lattice contraction. Moreover, the progressive broadening of the main diffraction peak with increasing Se content indicates enhanced lattice distortion and an elevated concentration of structural defects, which can significantly influence phonon scattering and thermal transport.

Further micrometer‐scale scanning electron microscope (SEM) imaging and energy‐dispersive X‐ray spectroscopy (EDS) elemental mapping (Figures S2 and S3) confirm the absence of secondary phase precipitates in both Bi_0.4_Sb_1.6_Te_3.01_ + 0.15% ZnSb and Bi_0.4_Sb_1.6_Te_2.97_Se_0.04_ + 0.15% ZnSb samples. All constituent elements exhibit uniform spatial distribution, consistent with the XRD results and indicating that both ZnSb and Se are predominantly incorporated into the Bi‐Sb‐Te matrix as solid‐solution species at the investigated doping levels. As shown in Figure S4, the microstructural porosity is significantly affected by ZnSb and Se alloying. For the ZnSb‐only incorporated samples (Figure S4a–d), the overall grain morphology remains essentially unchanged up to 0.2% ZnSb, while the density of micropores progressively decreases. This trend indicates that ZnSb incorporation effectively suppresses Te volatilization during sintering, thereby mitigating thermally induced pore formation and promoting a more compact microstructure. In contrast, increasing Se concentration (Figure S4e–g) leads to a more widespread distribution of micropores. This behavior is attributed to the higher intrinsic volatility of Se and its strong influence on defect chemistry, which enhances the formation of anion vacancies and related microvoids during synthesis.

**FIGURE 2:**
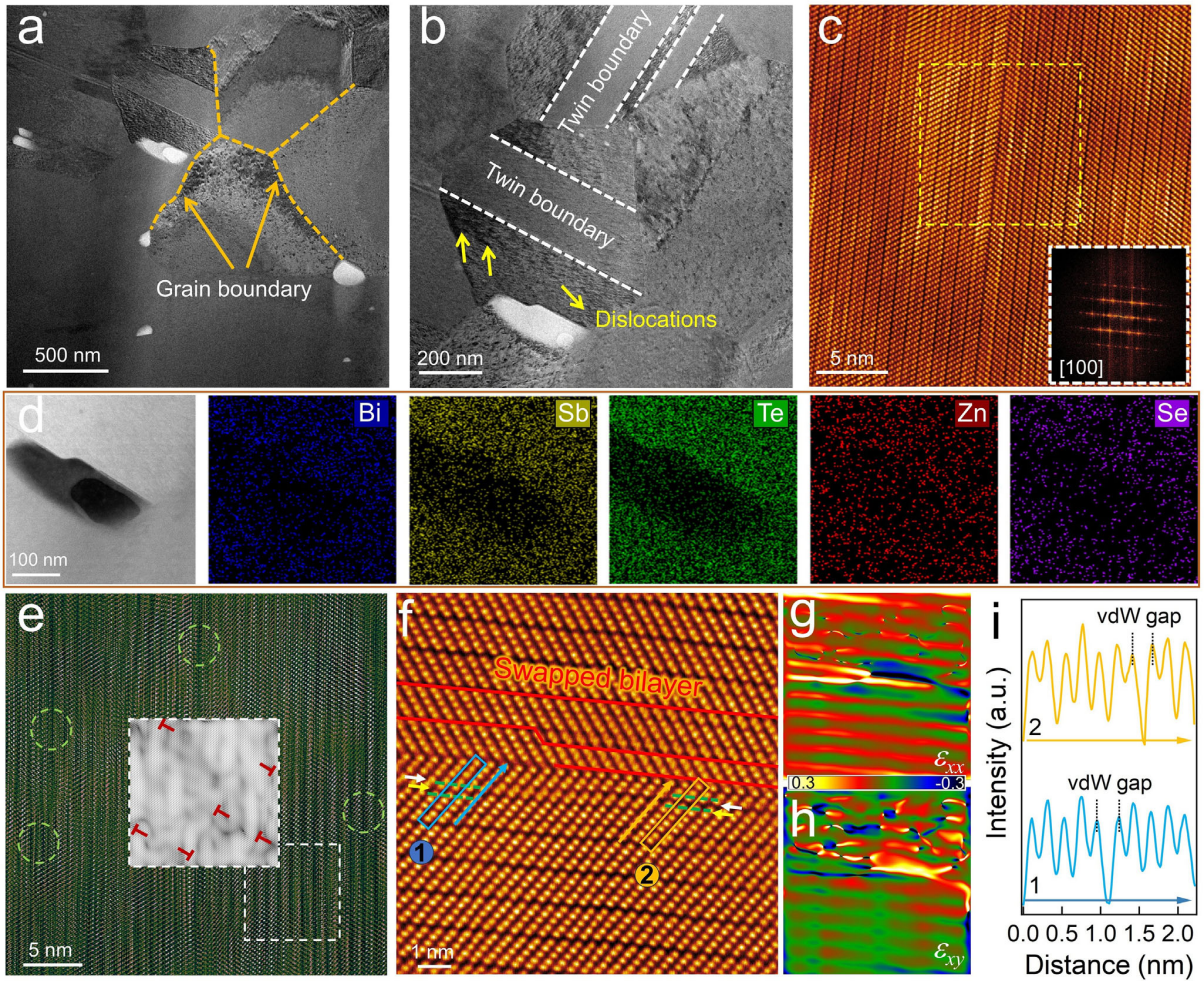
Microstructural characterization of Bi_0.4_Sb_1.6_Te_2.97_Se_0.04_ + 0.15% ZnSb sample. (a) Low‐magnification TEM image showing grain boundaries and micropores. (b) Low‐magnification TEM image highlighting TBs and dense dislocation networks. (c) HRTEM image of a representative TB, with the corresponding FFT pattern shown in the inset. (d) TEM‐EDS elemental mappings confirming the homogeneous distribution of Bi, Sb, Te, Zn, and Se. (e) HRTEM image revealing high densities of stacking faults (SFs) and dislocations. (f) HRTEM image displaying a characteristic SB structure (red‐lined region) adjacent to a TB. (g, h) GPA strain maps (*ε_xx_* and *ε_xy_*) corresponding to the SB‐containing region in (f), illustrating the localized lattice distortion. (i) Intensity line profiles extracted from the boxed regions 1 and 2 in (f), demonstrating the structural signatures of the SB configuration.

To clarify how nanoscale microstructural features regulate the TE properties, transmission electron microscopy (TEM) was conducted on the Bi_0.4_Sb_1.6_Te_2.97_Se_0.04_ + 0.15% ZnSb sample. The low‐magnification TEM image (Figure 2a) reveals well‐defined grain boundaries and randomly distributed submicron pores. TBs and dislocation arrays are identified within the grains (Figure 2b), indicating substantial defect accommodation induced by Se substitution and ZnSb incorporation. A representative coherent TB is shown in the high‐resolution TEM (HRTEM) image in Figure 2c. Such coherent interfaces introduce sharp phonon‐scattering centers while maintaining minimal perturbation to the electronic bands, thereby effectively reducing *κ*_l_ without significantly compromising *µ*_H_. TEM‐EDS elemental mappings collected at different magnifications around the pore regions (Figure 2d and Figure S5) exhibit a uniform distribution of all constituent elements, and no Zn‐rich clusters are detected, in contrast to the precipitation behavior reported in certain Zn‐based alloyed Bi_2_Te_3_ systems [27, 40]. As shown in Figure 2e, localized lattice‐stripe misalignments (highlighted by green dashed circles) indicate the presence of typical SFs. The corresponding inverse fast Fourier transform (IFFT) analysis further shows dislocations nucleated in close proximity to these SFs (marked by red symbols).

The atomic‐scale structure was further examined using phase‐corrected high‐angle annular dark‐field (HAADF) imaging, in which the contrast varies approximately with the square of the atomic number (Figure 2f). Distinct atomic arrangements resembling the recently reported SB are clearly observed near the TB, as highlighted by the red guidelines [41]. Geometric phase analysis (GPA) (Figure 2g,h) reveals significant strain localization at the SB/TB interface, along with a redistribution of strain fields across the adjacent vdW gaps. Such strain coupling reflects the strong interaction between defect‐driven structural distortion and the layered host lattice. The line‐intensity profiles extracted from regions 1 and 2 in Figure 2f (Figure 2i) confirm a local structural evolution from the native quintuple‐layer (QL) sequence toward a septuple‐layer (SL) configuration. This transformation indicates that Se doping promotes the formation of 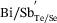 antisite defects. When these antisite defects align continuously along one side of the vdW gap, they form distorted Bi/Sb‐Te/Se bonding networks that couple adjacent QLs, thereby generating localized SL units. Meanwhile, the TB—characterized by a low interfacial energy—serves as an efficient kinetic pathway for atomic sliding along the (00*l*) orientation [41, 42, 43]. The cooperative action between this sliding mechanism and antisite‐defect‐mediated layer reconstruction facilitates the gradual reorganization of the layered stacking and ultimately stabilizes the SB structure.

**FIGURE 3:**
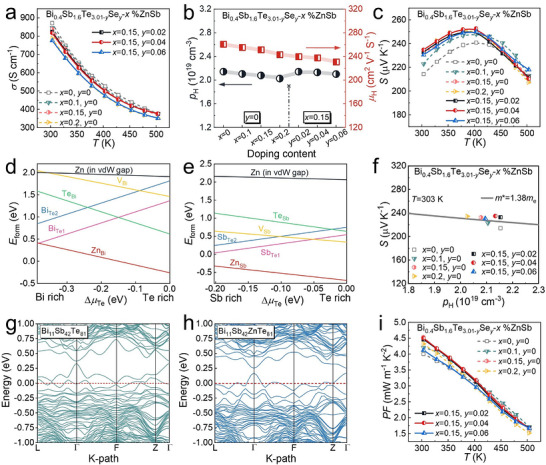
Electronic transport properties of Bi_0.4_Sb_1.6_Te_3.01‐_ *_y_* Se*_y_* + *x*% ZnSb samples. (a) Temperature‐dependent *σ*, (b) carrier concentration (*p*_H_) and Hall mobility (*µ*_H_) determined from Hall measurements at 303 K, and (c) *S* as a function of temperature. (d, e) Theoretically calculated defect formation energies in (d) Bi_2_Te_3_ and (e) Sb_2_Te_3_, including spin‐orbit coupling effects. (f) Pisarenko plot correlating *p*_H_ and *S* at 303 K. (g, h) Electronic band structures of (g) Bi_11_Sb_42_Te_81_ and (h) Bi_11_Sb_42_ZnTe_81_, illustrating the effect of Zn incorporation. (i) Temperature‐dependent *PF* of Bi_0.4_Sb_1.6_Te_3.01‐_ *_y_* Se*_y_* + *x*% ZnSb.

Figure 3 presents the influence of ZnSb and Se incorporation on the electrical transport properties of the Bi‐Sb‐Te system. As shown in Figure 3a, the *σ* of all samples decreases monotonically with increasing temperature, characteristic of degenerate semiconductors. A pronounced decline in *σ* is observed with higher ZnSb content, and subsequent Se incorporation further suppresses *σ*. Room‐temperature Hall measurements (Figure 3b) indicate that this reduction originates from simultaneous decreases in *p*_H_ and *µ*_H_. Notably, at low Se doping levels (*x* = 0.15, *y* = 0.02), a slight increase in *p*_H_ is detected. This behavior is attributed to the enhanced formation of 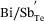 antisite defects. As shown in Figure 3c, S exhibits an overall enhancement upon ZnSb and Se incorporation. This improvement stems from the effect of reduced *p*_H_.

To elucidate the origin of the reduced *p*_H_, defect formation energy calculations were performed for Bi_2_Te_3_ and Sb_2_Te_3_ under Te‐rich conditions (Figure 3d,e). Owing to the prohibitively high formation energy of Zn incorporation into the vdW gap and the uniformly distributed elemental signals revealed by SEM/TEM‐EDS mapping, Zn is more likely to occupy Sb sites (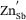), while antisite defects (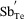) and Sb atoms filling cation vacancies are also present as dominant defect configurations. Notably, the reduction in *p*_H_ is primarily attributed to the suppression and compensation of intrinsic cation vacancies by Sb filling, which effectively decreases the hole concentration. In contrast, although 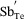 and 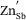 defects introduce holes from the perspective of defect charge states, their contributions are relatively secondary and mainly associated with the modulation of the overall defect landscape. From the defect chemistry perspective, each Sb‐filling cation vacancy introduces three electrons, whereas 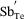 and 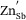 defects contribute to two holes. The competition among these defects results in a net decrease in *p*_H_, with vacancy compensation playing the dominant role.

The Pisarenko relation (*p*_H_‐*S*) at 303 K shows that the effective mass of the doped samples remains essentially unchanged within the experimental uncertainty (m*∼1.38 *m*_e_), indicating that ZnSb and Se incorporation exert minimal influence on the intrinsic band curvature of the Bi‐Sb‐Te host (Figure 3f). This observation is corroborated by density functional theory (DFT) calculations (Figure 3g,h), which reveal that ZnSb incorporation causes negligible perturbation to the overall band structure. However, the noticeable upward shift of the Fermi level toward the valence band maximum (VBM) provides strong evidence for the experimentally observed reduction in *p*_H_. The corresponding total and partial density of states (DOS) profiles (Figure S6a,b) further validate this Fermi level shift. Moreover, the incorporation of additional Se results in only minor variations in the band structure (Figure S6c,d). Benefiting from the enhanced *S* in the doped samples, the *PF* shows a clear improvement. In particular, ZnSb incorporation substantially enhances the *PF*, while the co‐doped sample with optimal Se concentration (*x* = 0.15, *y* = 0.04) maintains a comparably high *PF* across a broad temperature range.

**FIGURE 4:**
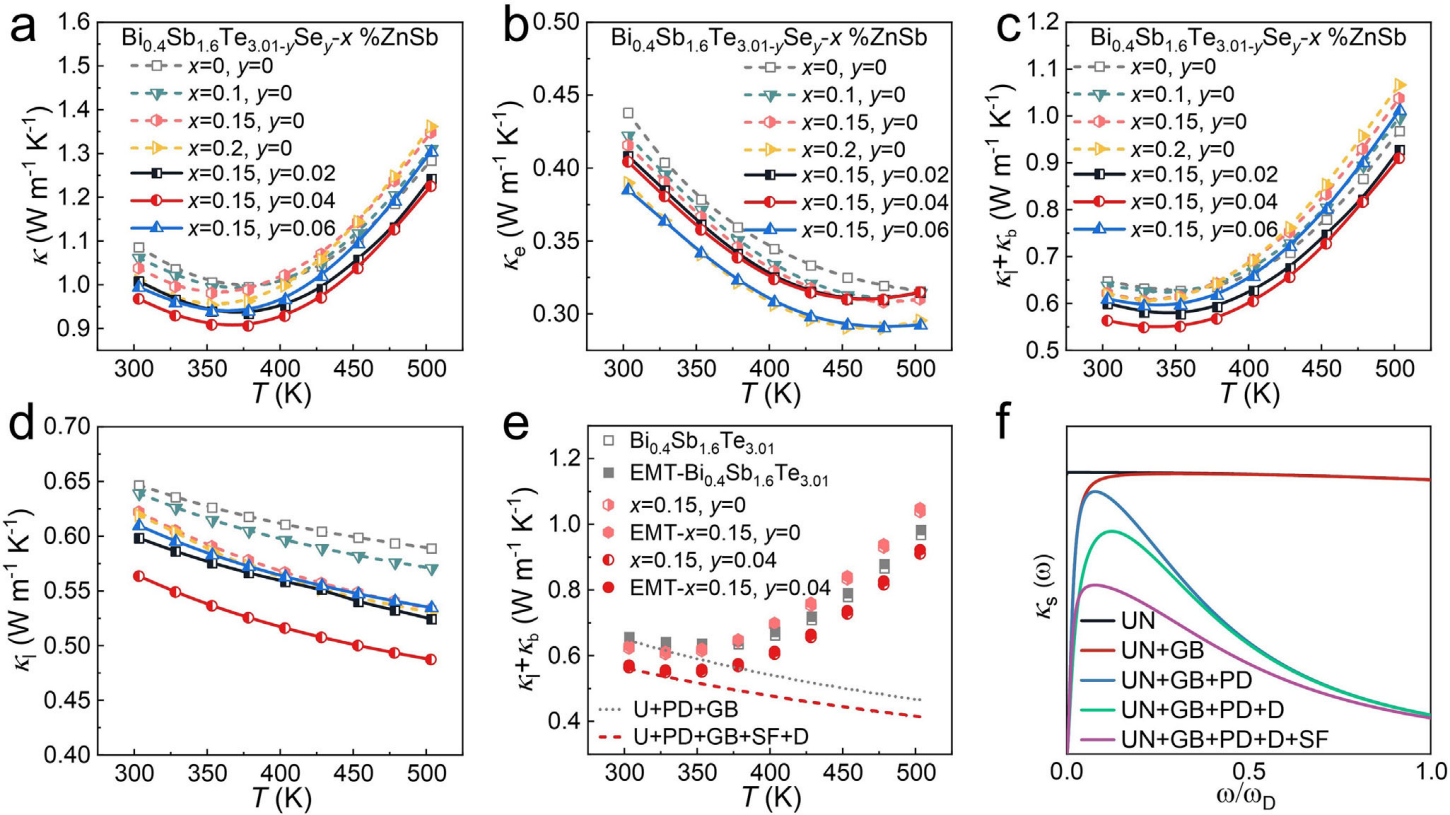
Thermal transport properties of Bi_0.4_Sb_1.6_Te_3.01‐_ *_y_* Se*_y_* + *x*% ZnSb samples. (a) Temperature‐dependent (a) *κ*, (b) *κ*_e_, (c) *κ*_l_ + *κ*_b_, and (d) *κ*_l_. (e) Comparison between experimental *κ*_l_ + *κ*_b_ and theoretical values calculated using the Debye‐Callaway model (bipolar diffusion excluded). (f) Frequency‐dependent spectral lattice thermal conductivity (*κ*_s_) of Bi_0.4_Sb_1.6_Te_3.01_.

Figure 4 presents the thermal transport properties of the Bi_0.4_Sb_1.6_Te_3.01‐_ *_y_* Se*_y_* + *x*% ZnSb samples. The combined introduction of ZnSb and Se leads to a pronounced reduction of *κ* (Figure 4a). As shown in Figure 4b–d, slight ZnSb incorporation predominantly decreases *κ*_e_, while exerting only a modest influence on *κ*_l_. According to the Wiedemann‐Franz relation, *κ*_e_ = *LσT*, where the Lorentz number *L* can be estimated using *L* = (1.5 + exp(−|*S*|/116)) × 10^−8^ V^2^ K^−2^. This reduction originates mainly from Sb partially occupying the cation vacancies, thereby introducing additional carrier scattering centers that significantly reduce *µ*_H_, leading to suppressed *σ* and *κ*_e_.

For Bi_2_Te_3_‐based TE systems, bipolar conduction must be considered when evaluating *κ*_l_. At low temperatures, the contribution from *κ*_b_ is negligible. It's assumed to dominate and follow an approximate 1/*T* behavior above the Debye temperature. Using *κ*_l_ = *aT* ^−1^ + *b*, the *κ*_l_ value before bipolar conduction begins can be estimated and extrapolated to higher temperatures [8, 44]. While ZnSb incorporation mainly suppresses *κ*_e_, subsequent Se doping exerts a more substantial influence on *κ*_l_. This enhancement in phonon scattering arises from two primary mechanisms. First, the large atomic mass disparity between Se and Te strengthens point‐defect scattering of mid‐to‐high‐frequency phonons through mass‐fluctuation‐induced perturbations. Second, the high volatility of Se increases the concentration of 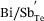 antisite defects, and the associated lattice distortion and local strain fields further impede phonon propagation. The Debye–Callaway model was employed to evaluate the contributions of multiple phonon scattering mechanisms in TE materials [45, 46, 47].

(1) $$\kappa_{l}=\frac{k_{B}}{2\pi^{2}v}\;{\left(\frac{k_{B}T}{\hbar }\right)}^{3}\int_{0}^{\theta_{D}/T}\tau_{\mathrm{tot}}(x)\frac{x^{4}e^{x}}{{\left(e^{x}-1\right)}^{2}}dx$$

Here, *k*_B_, *ћ*, *v*, *T*, and *θ*_D_ denote the Boltzmann constant, reduced Planck constant, average sound velocity, absolute temperature, and Debye temperature, respectively, and *x* = ℏω/*k*_B_ *T* represents the simplified phonon frequency. As shown in Figure 4e, the experimentally obtained *κ*_l_ + *κ*_b_ values were corrected using effective medium theory (EMT) [48, 49, 50, 51]. Given the small and uniformly distributed pore sizes, the porosity remains low, resulting in minimal deviation between EMT‐corrected and measured *κ*_l_ + *κ*_b_. When additional phonon scattering channels—namely dislocations (D) and stacking‐faults (SF, including SB)–related interface scattering—are incorporated alongside intrinsic Umklapp (U) and Normal (N) processes, point‐defect (PD) scattering, and grain‐boundary (GB) scattering, the overall *κ*_l_ is evidently suppressed, as reflected by the red dashed curve in Figure 4e. The frequency‐dependent spectral lattice thermal conductivity (*κ*_s_) in Figure 4f further elucidates the phonon regimes dominated by different scattering mechanisms. By introducing ZnSb and Se, vacancy filling together with 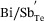 and Se_Te_ point defects dominantly scatter high‐frequency phonons. The high‐density dislocation arrays and SFs effectively disrupt mid‐ and low‐frequency phonon transport.

**FIGURE 5:**
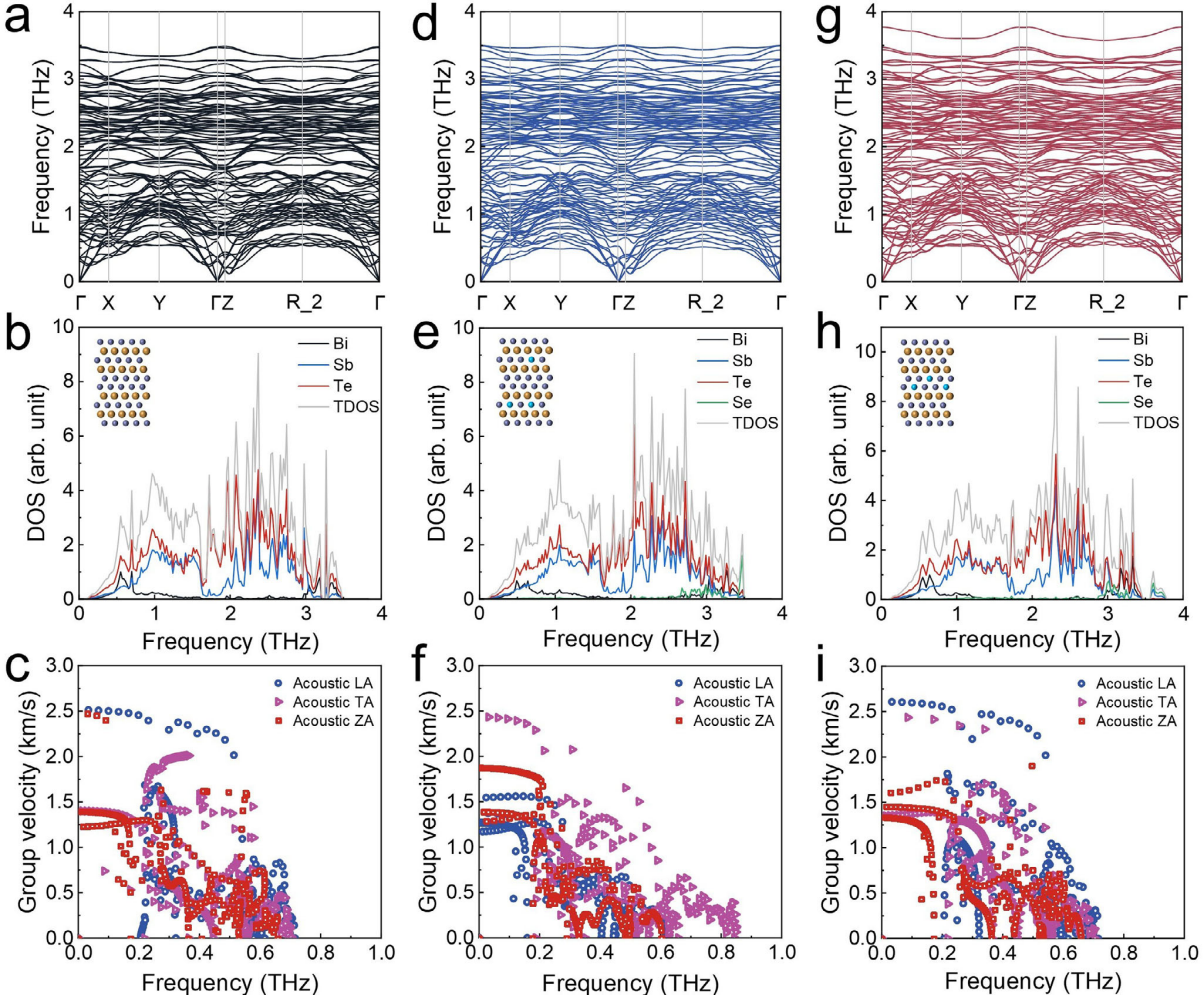
Calculated phonon dispersion of Bi‐Sb‐Te‐Se systems. (a–c) Phonon dispersion curves, phonon DOS, and phonon group velocities of Bi_2_Sb_10_Te_18_. (d–f) Corresponding phonon dispersion, phonon DOS, and phonon group velocities of Bi_2_Sb_10_Te_17_Se, where Se substitutes for Te within the QL. (g–i) Phonon dispersion, phonon DOS, and phonon group velocities of Bi_2_Sb_10_Te_17_Se with Se occupying Te sites located in the interlayer vdW gaps.

To elucidate the mechanisms underlying the enhanced phonon scattering, phonon dispersion calculations were performed. Considering the relatively low Se content and experimental observability of the phonon spectra, a 30‐atom supercell was adopted for qualitative analysis [52, 53, 54, 55]. Figure 5a–c present the phonon transport characteristics of the pristine system. As shown in Figure 5a, phonon dispersion originates from zero frequency and extends along the high‐symmetry directions, displaying typical acoustic branches that gradually bend and flatten near the Brillouin zone boundaries. Figure 5b shows that Te atoms contribute across the entire frequency range due to their involvement in lattice vibrations; however, they dominate the mid‐to‐high‐frequency optical modes because of their relatively light mass and strong localized bond‐stretching vibrations. In contrast, the low‐frequency acoustic modes mainly arise from the coupled motions of heavier Bi/Sb cations and Te anions. As illustrated in Figure 5c, the longitudinal (LA) and transverse (TA) acoustic branches maintain relatively high group velocities, contributing predominantly to *κ*_l_, whereas the out‐of‐plane (ZA) mode exhibits a lower velocity due to the weak vdW interlayer forces, making only a minor contribution to *κ*_l_.

A clear site‐dependent effect is observed upon Se incorporation. When Se substitutes Te within the QL, partial flattening of the dispersion curves is observed (Figure 5d–f). Additional mid‐to‐high‐frequency states appear near the Se sites, stemming from the lighter mass of Se and localized bonding effects. The softening (downshift) of the acoustic phonons reflects a reduction in the interatomic force constants. Correspondingly, the group velocities within the 0.2–0.6 THz range are substantially reduced, implying that the long‐range propagation of acoustic phonons is strongly hindered by bond disorder and increased scattering. Consequently, the disruption of the lattice periodicity substantially weakens acoustic phonon transport, leading to a significant reduction in *κ*_l_. In contrast, when Se occupies Te sites located in the interlayer vdW gaps (Figure 5g–i), the phonon dispersion relations and acoustic branch velocities remain nearly indistinguishable from those of the pristine lattice. This indicates that the long‐range crystallographic order is largely preserved, allowing heat‐carrying acoustic phonons to propagate efficiently. Consequently, no substantial degradation of *κ*_l_ is expected for Se substitution at Te sites located in the interlayer vdW gaps.

**FIGURE 6:**
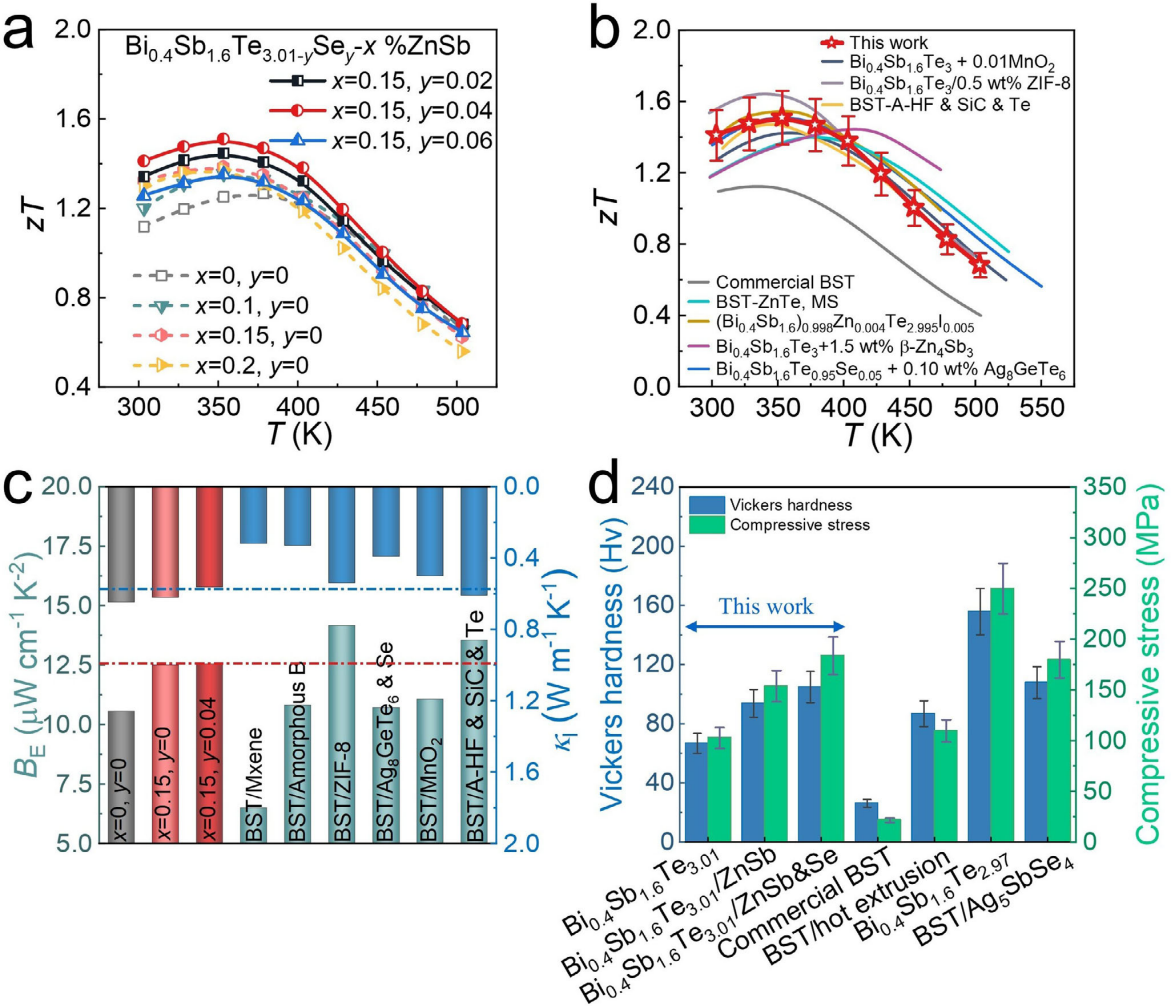
The *zT* values, electrical and thermal transport analysis for quality factor and mechanical properties of Bi_0.4_Sb_1.6_Te_3.01‐_ *_y_* Se*_y_* + *x*% ZnSb samples. (a) Temperature‐dependent *zT* values of the Bi_0.4_Sb_1.6_Te_3.01‐_ *_y_* Se*_y_* + *x*% ZnSb samples. (b) Comparison of the *zT* values obtained in this work with high‐performance p‐type (Bi, Sb)_2_Te_3_‐based materials reported in the literature [27, 29, 32, 38, 39, 56, 57]. (c) Electrical quality factor (*B*_E_) compared with other recent representative studies, accompanied by the corresponding *κ*_l_ values [27, 29, 32, 39, 58, 59]. (d) Comparison of Vickers hardness and compressive stress for pristine Bi_0.4_Sb_1.6_Te_3.01_, Bi_0.4_Sb_1.6_Te_3.01_ + 0.15% ZnSb, and Bi_0.4_Sb_1.6_Te_2.97_Se_0.04_ + 0.15% ZnSb, benchmarked against representative mechanically robust (Bi, Sb)_2_Te_3_‐based materials [60, 61, 62, 63].

Following Se doping, the Bi_0.4_Sb_1.6_Te_2.97_Se_0.04_ + 0.15% ZnSb sample achieves a peak *zT* of ∼1.51 at 353 K, benefiting from the concurrent preservation of a high *PF* and a substantial reduction in *κ*_l_ (Figure 6a). The reproducibility of the TE performance is confirmed using independently re‐synthesized samples (Figure S7). To further assess the intrinsic anisotropy of (Bi, Sb)_2_Te_3_, transport measurements were performed perpendicular to the hot‐pressing direction (Figure S8). As shown in Figure 6b, benchmarked against several state‐of‐the‐art doping and composite strategies reported in recent literature, the present system delivers superior *zT* across the entire measured temperature range [27, 29, 32, 38, 39, 56, 57]. Notably, *zT*_ave_ reaches ∼1.47 below 403 K, effectively overcoming the inherent low‐temperature limitations of commercial p‐type Bi‐Sb‐Te materials. This high performance enables the material to serve dual functional roles in both solid‐state refrigeration and TE power generation. The TE quality factor (*B*) determines *zT* and peak *zT* of a material, thereby offering insights into performance optimization. Defined as *B* = *B*_E_ *T*/*κ*_l_, where *B*_E_ denotes the electronic quality factor. As shown in Figure 6c, a comparison of *B*_E_ and *κ*_l_ with representative systems reported in recent years confirms that Bi_0.4_Sb_1.6_Te_2.97_Se_0.04_ + 0.15% ZnSb retains substantial headroom for further optimization [27, 29, 32, 39, 58, 59].

Figure 6d illustrates that ZnSb incorporation effectively strengthens the local bonding environment by occupying cation vacancies. In parallel, Se substitution induces an SB configuration that enhances interlayer coupling and lattice distortion. The cooperative action of these two mechanisms significantly improves crack resistance and plastic deformability, as reflected by the pronounced increases in both Vickers hardness and compressive strength. The optimized samples demonstrate markedly enhanced mechanical robustness, outperforming several recently reported high‐performance TE materials [33, 60, 61, 62].

**FIGURE 7:**
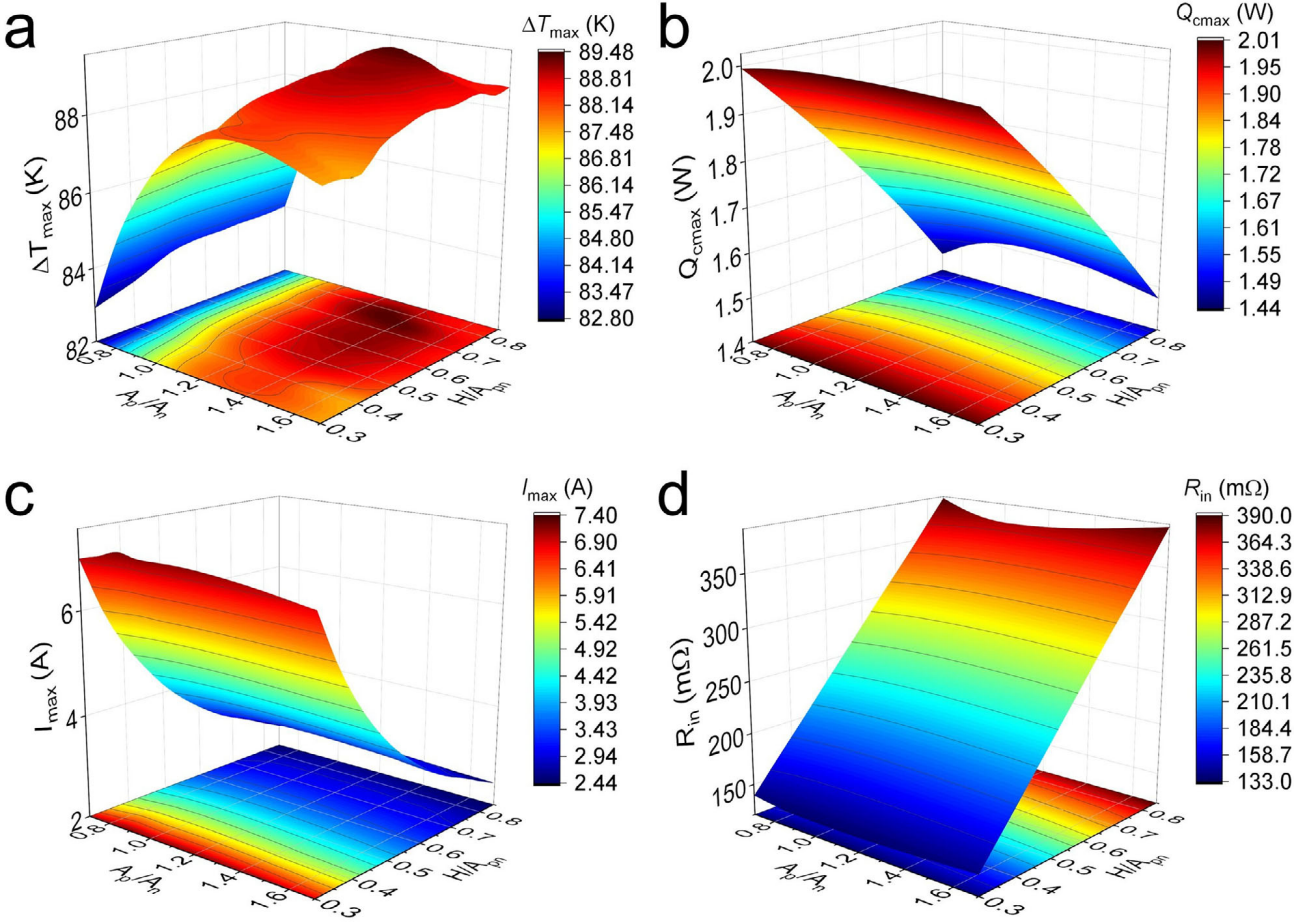
Theoretical simulation for TE devices. Finite‐element‐simulated three‐dimensional (3D) distributions of (a) the maximum cooling temperature difference (Δ*T*_max_), (b) maximum cooling capacity (*Q*_cmax_), (c) maximum operating current (*I*_max_) corresponding to Δ*T*_max_, and (d) internal resistance (*R*_in_) of the TE device at a fixed hot‐side temperature (*T*_h_) of 303 K. The 3D contour maps are plotted as functions of the geometric parameters *H*/*A*_pn_ and *A*_p_/*A*_n_, where *H*, *A*_p_, and *A*_n_ represent the leg height and the cross‐sectional areas of the p‐type and n‐type legs, respectively (*A*_pn_ = *A*_p_ + *A*_n_).

To capitalize on the excellent near‐room‐temperature TE performance, finite element analysis (FEA) was carried out using the experimentally measured transport parameters of Bi_0.4_Sb_1.6_Te_2.97_Se_0.04_ + 0.15% ZnSb and Bi_2_Te_2.7_Se_0.3_ (Figure S9) to optimize device geometry for cooling at 303 K. The boundary conditions used in the FEA simulations are detailed in the Supporting Information. The model consists of a device with seven p‐n couples assembled on a 10 mm × 10 mm Cu‐clad Al_2_O_3_ substrate. Here, *A*_p_, *A*_n_, *H*, Δ*T*_max_, *Q*_cmax_, *I*_max_, and *R*_in_ represent the cross‐sectional areas of the p‐type and n‐type legs, leg height, maximum achievable temperature difference, maximum cooling capacity, current at Δ*T*_max_, and internal resistance, respectively. As shown in Figure 7a, Δ*T*_max_ increases with increasing *H*/*A*_pn_, because longer legs effectively suppress thermal backflow and sustain a larger temperature gradient. However, the accompanying rise in *R*_in_ leads to substantial Joule heating at high *H*/*A*_pn_, resulting in the reduction of *Q*_cmax_ (Figure 7b). In contrast, decreasing *H*/*A*_pn_ lowers *R*_in_ and boosts *I*_max_, thereby enhancing *Q*_cmax_. Nevertheless, this condition limits the attainable Δ*T*_max_ (Figure 7c,d). These competing trends underscore the intrinsic trade‐off between Δ*T*_max_ and *Q*_cmax_, necessitating a balanced geometric design for optimal device performance. Through comprehensive FEA optimization, the ideal configuration was identified at *H*/*A*_pn_ = 0.65 and *A*_p_/*A*_n_ = 1.3, corresponding to p‐type and n‐type leg dimensions of 1.6 mm × 1.6 mm × 3 mm and 1.4 mm × 1.4 mm × 3 mm, respectively.

**FIGURE 8:**
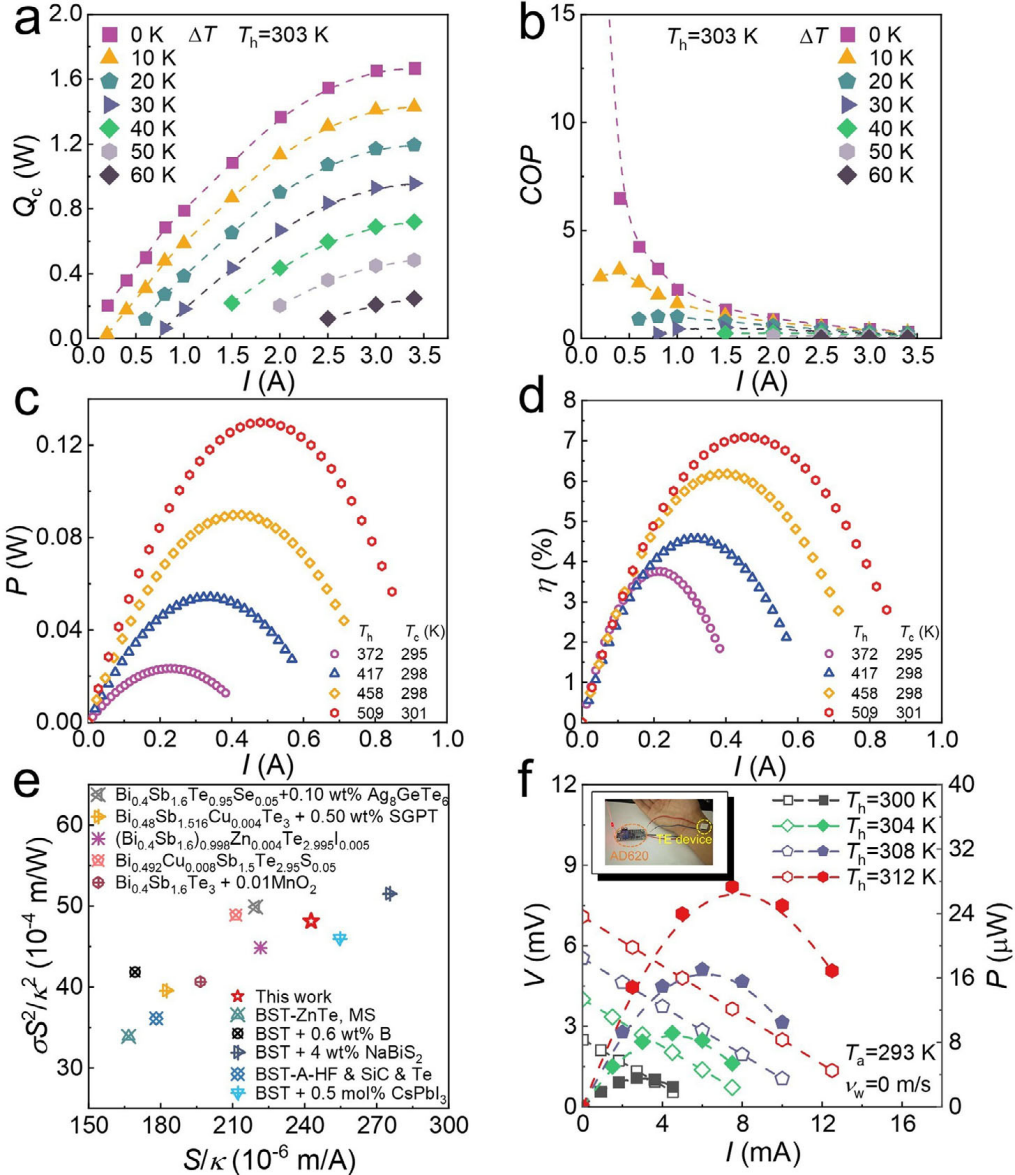
Cooling and power‐generation performance for fabricated TE devices. At a fixed *T*_h_ of 303 K, (a) measured cooling capacity (*Q*_c_) and (b) coefficient of performance (COP) as functions of *I*. Under various *T*_h_ and *T*_c_ conditions, (c) output power (*P*) and (d) *η* as functions of *I*. (e) Correlation between *σS* ^2^/*κ* ^2^ and *S*/*κ* for representative p‐type Bi_2_Te_3_‐based materials. Here, MS denotes melt spinning, and A‐HF refers to the combined annealing‐hot‐forging process [27, 30, 32, 33, 36, 38, 39, 59, 64]. (f) Measured *V* and *P* as functions of *I* at different *T*_h_ values. The inset shows a wrist‐worn TE device operating in a wind‐free chamber, powering an LED through voltage amplification.

Guided by the FEA‐derived optimal geometry, TE devices were fabricated and assembled to experimentally validate their performance. A schematic of the TE cooling measurement system is provided in Figure S10. As shown in Figure 1b(b1), the experimentally measured cooling Δ*T* at *T*_h_ = 303 K follows the trend predicted by simulation, with the deviation mainly arising from the limited vacuum level of the test chamber, parasitic electrical losses, interfacial resistance/thermal resistance, and dimensional tolerances during leg machining. As the current (*I*) increases toward *I*_max_, Δ*T* reached Δ*T*_max_ ∼70 K. Beyond this point, Δ*T* gradually declines with further increases in *I*, which was mainly caused by the intensified Joule heating. Figure 8a demonstrates that the cooling capacity (*Q*_c_) exhibits a positive correlation with both *I* and Δ*T*. At Δ*T* = 0 K, *Q*_cmax_ reaches 1.67 W at *I* = 3.4 A, while the maximum coefficient of performance (COP_max_) attains 6.5 at *I* = 0.4 A (Figure 8b).

Beyond its outstanding cooling performance, the Bi_0.4_Sb_1.6_Te_2.97_Se_0.04_ + 0.15% ZnSb/Bi_2_Te_2.7_Se_0.3_ TE device also exhibits excellent power generation capability. The linear voltage–current (*V*–*I*) characteristics (Figure S11a) agree well with ideal circuit behavior. As shown in Figure 8c, the output power (*P*) increases with *I* and reaches a maximum (*P*_max_ = 0.13 W) under the condition that the *R*_in_ equals the load resistance at *T*_h_‐*T*_c_ = 208 K. Owing to the reduced heat flux (*Q*) enabled by the intrinsically low *κ* of the materials (Figure S11b), the compact 10 mm × 10 mm device containing seven p‐n couples achieves an ultra‐high maximum power conversion efficiency (*η*_max_) of ∼7.1% at *T*_h_‐*T*_c_ = 208 K (Figure 8d). As highlighted in Figure 1b(b2), the demonstrated performance surpasses several representative Bi‐Sb‐Te‐based power generation devices reported in recent literature [29, 32, 33, 36, 37, 38, 39].

Furthermore, the fabricated device maintains highly competitive output performance under practical conditions relevant to wearable electronics. The maximum open‐circuit voltage density (*V*_oc_/*A*) and maximum power density (*P*_max_/*A*) can be approximated as [65]:

(2) $$V_{\mathrm{oc}}/A=\frac{\left|S\right|h_{a}H}{A\left(h_{a}H+\kappa \right)}\left(T_{h}-T_{a}\right)$$

(3) $$P_{\max}/A=\frac{\sigma S^{2}}{{\left(h_{a}H+\kappa \right)}^{2}}\frac{{\left(T_{h}-T_{a}\right)}^{2}{h_{a}}^{2}H}{4}$$

where *A*, *h*_a_ and *T*_a_ denote the cross‐sectional area of the device, heat transfer coefficient between the cold‐side leg surface and ambient air, and ambient temperature, respectively. Under fixed geometric parameters (*A*, *H*) and boundary conditions (*h*_a_, *T*_h_, *T*_a_), a larger *S/κ* ratio of the TE legs leads directly to enhanced *V*_oc_/*A* and *P*_max_/*A*. As shown in Figure 8e, the material developed in this work demonstrates a distinctly superior power density potential compared with several high‐performance p‐type Bi_2_Te_3_‐based TE materials, underscoring its strong applicability for wearable TE harvesting [27, 30, 32, 33, 36, 38, 39, 59, 64]. Figure 1b(b3) shows the experimentally measured *V*_oc_/*A* and *P*_max_/*A* under fixed *T*_a_ across different *T*_h_. Both parameters increase monotonically with rising *T*_h_, reaching a *P*_max_/*A* of 27.3 µW cm^−2^ at *T*_h_ = 312 K and *T*_a_ = 293 K under windless conditions (*v*_w_ = 0 m/s). Figure 8f further displays the measured *V* and *P* as functions of *I*, confirming the consistent operational stability of the device. The inset photograph illustrates a wearable TE device powering an LED through voltage amplification using an AD620 operational amplifier, demonstrating its practical feasibility for low‐power wearable electronics.

## 3 Conclusions

In summary, we demonstrate a concise yet powerful dual‐regulation strategy that integrates trace intermetallic ZnSb incorporation with Se doping to synergistically optimize TE performance in p‐type Bi_2_Te_3_, while enabling multifunctional device applications through optimized module design. Notably, ZnSb incorporation primarily regulates carrier transport via defect engineering, particularly through Sb vacancy compensation, whereas Se doping mainly modulates lattice dynamics by enhancing phonon scattering and lattice anharmonicity. This functional differentiation avoids redundancy and enables a complementary synergistic effect. ZnSb incorporation effectively compensates Sb vacancies and suppresses Te volatilization, thereby reducing *p*_H_ and enhancing *S*. Concurrently, Se doping refines lattice dynamics by introducing multiple phonon scattering centers and induces the formation of SB structures near TBs, strengthening interlayer coupling and improving structural robustness. Driven by these cooperative effects, the optimized Bi_0.4_Sb_1.6_Te_2.97_Se_0.04_ + 0.15% ZnSb sample achieves a high peak *zT* of ∼1.51 at 353 K and a *zT*_ave_ of ∼1.47 below 403 K, accompanied by remarkable mechanical reliability (Vickers hardness ∼97 Hv; compressive strength ∼188 MPa). The FEA simulation further verifies that a 7‐pair TE device constructed from this material delivers a large Δ*T*_max_ of ∼70 K at 303 K and an ultra‐high *η*_max_ of ∼7.1% at *T*_h_‐*T*_c_ = 208 K, while sustaining competitive output performance under ambient wearable conditions. This work bridges atomic‐scale defect manipulation with device‐level optimization, offering a universal paradigm for developing multifunctional TE systems that simultaneously achieve high efficiency and mechanical durability. The proposed strategy provides a practical and scalable route toward next‐generation near‐room‐temperature TE materials with promising applications in solid‐state cooling, low‐grade heat harvesting, and wearable electronics.

## Conflicts of Interest

The authors declare no conflicts of interest.

## Supporting information




**Supporting File**: advs75529‐sup‐0001‐SuppMat.docx.

## Data Availability

The data that support the findings of this study are available from the corresponding author upon reasonable request.
