# Supplementary material for: Coupled Vacancy and Phonon‐Scattering Engineering Drive Defect Evolution Toward Multifunctional High‐Performance Bi2Te3 Thermoelectrics
Source: Adv Sci (Weinh). 2026 May 4;13(42):e75529. doi: 10.1002/advs.75529 (PMC13335580; doi:10.1002/advs.75529)
Supplement: Supplementary file 1 — Supporting File: advs75529‐sup‐0001‐SuppMat.docx. [file ADVS-13-e75529-s001.docx]

**Supplementary materials**

**Coupled Vacancy and Phonon-Scattering Engineering Drive Defect Evolution toward Multifunctional High-Performance Bi_2_Te_3_ Thermoelectrics**

Ruiheng Li, Minwen Yang, Huangshui Ma, Jie Zheng, Shengqiang Cui, Xiaobo Tan, Xuri Rao, Xiang An, Zongxiang Kan, Siqi Huo, Jing Shuai*, Min Hong*, and Ran Ang*

R. H. Li, J. Zheng, S. Q. Cui, X. B. Tan, X. R. Rao, X. An, R. Ang

Key Laboratory of Radiation Physics and Technology, Ministry of Education, Institute of Nuclear Science and Technology, Sichuan University, Chengdu 610064, China

E-mail: [rang@scu.edu.cn](mailto:rang@scu.edu.cn)

M. W. Yang, J. Shuai

School of Materials Sun Yat-sen University, Shenzhen 518107, China

E-mail: [shuaij3@mail.sysu.edu.cn](mailto:shuaij3@mail.sysu.edu.cn)

Z. X. Kan

No. 26 Jingbei Road, Xianghe Environmental Protection Industrial Park, Xianghe 065400, China

H. S. Ma, S. Q. Huo, M. Hong

Centre for Future Materials, School of Science, Engineering and Digital Technologies, University of Southern Queensland, Springfield Campus, QLD 4300, Australia

E-mail: min.hong@unisq.edu.au

R. Ang

Institute of New Energy and Low-Carbon Technology, Sichuan University, Chengdu 610065, China

College of Physics, Sichuan University, Chengdu 610064, China

Keywords: Bi_2_Te_3_-based materials; vacancy compensation; phonon scattering; swapped bilayer structures; multifunctional thermoelectric devices

**Supplementary Methods**

1. **Sample synthesis**

High-purity Zn (granules, 5N, Aladdin) and Sb (granules, 5N, Aladdin) were weighed according to the stoichiometric ratio of ZnSb. The elements were loaded into quartz ampoules, evacuated to ~10^-3^ Torr, and flame-sealed. The sealed ampoules were heated to 1023 K, held for 24 h to ensure complete reaction, and subsequently quenched in ice water. The obtained ingots were then annealed at 573 K for 48 h to achieve phase homogenization.

For the preparation of Bi_0.4_Sb_1.6_Te_3.01-_*_y_*Se*_y_* + *x*% ZnSb (*x* = 0, 0.1, 0.15, 0.2; *y*=0.02, 0.04, 0.06), pre-synthesized ZnSb and high-purity elemental Bi (granules, 4N, Aladdin), Sb (granules, 5N, Aladdin), Te (chunks, 5N, Aladdin), and Se (granules, 5N, Aladdin) were weighed and mixed according to the target compositions. The mixtures were sealed in evacuated quartz ampoules and heated to 1073 K with a ramping time of ~5 h ramping, kept at this temperature for 6 h, and then slowly furnace-cooled to room temperature over >10 h.

The solidified ingots were transferred to stainless-steel milling jars inside an argon-filled glovebox and mechanically milled at 1800 rpm for 30 min. The resulting fine powders were loaded into Ø12.7 mm graphite dies and consolidated via hot pressing at 693 K for 15 min under a uniaxial pressure of 50 MPa and a vacuum below 5 Pa. All consolidated pellets exhibited relative densities greater than 95% of their theoretical values.

1. **Structural and mechanical characterization**

The phase purity and crystal structure of the samples were examined by X-ray diffraction (XRD) using Cu Kα radiation. The morphology and elemental distribution were characterized by a scanning electron microscope (SEM, FEI Inspect F50) equipped with an energy-dispersive X-ray spectroscopy (EDS) system for elemental mapping. Transmission electron microscopy (TEM, Philips Tecnai F20), operated in conjunction with EDS, was employed to analyze the microstructure, defect features, and local chemical compositions. Microhardness measurements were performed using a Vickers diamond indenter (HVS-1000) under a load of 2 N with a dwell time of 10 s. The compressive stress-strain behavior of the bulk samples was evaluated using a universal testing machine at a constant loading rate of 0.5 mm/min.

1. **Thermoelectric (TE) property measurements**

Electrical transport properties were measured using a CTA Pro system (Beijing Cryoall Science and Technology Co., Ltd.). Thermal diffusivity (*D*) was obtained by the laser flash method using a NETZSCH LFA-467 HT instrument. The total thermal conductivity (*κ*) was calculated as *κ* = *D* × *C*_p_ × *r*, where *C*_p_ (specific heat) was estimated using the Dulong–Petit law and *r* (geometrical density) was determined via the Archimedes method. Hall mobility (*m*_H_) and carrier concentration (*p*_H_) were evaluated by the van der Pauw method under a reversible magnetic field of 1.5 T. All electrical and thermal transport measurements were carried out parallel to the uniaxial hot-pressing (HP) direction. The estimated measurement uncertainties were ± 2% for electrical conductivity (*σ*), ± 5% for the Seebeck coefficient (*S*), and ± 3% for *κ*, yielding an overall uncertainty of ± 10% for *zT*.

1. **Computational methods**

Density functional theory (DFT) calculations were performed using the Vienna ab initio simulation package (VASP) with VASPKIT for post-processing [1-4]. The exchange-correlation interactions were treated using the Perdew-Burke-Ernzerhof (PBE) functional within the generalized gradient approximation (GGA) [5]. A 3 × 3 × 3 supercell based on the rhombohedral Sb_2_Te_3_ primitive cell was constructed. Ternary alloying was modeled by substituting 11 Sb atoms with Bi to form Bi_11_Sb_43_Te_81_. Sb vacancies and Zn substitution were introduced by removing Sb atoms or replacing Sb with Zn, generating Bi_11_Sb_42_Te_81_ and Bi_11_Sb_42_ZnTe_81_, respectively. Se doping was simulated by replacing one Te atom with Se, yielding Bi_11_Sb_42_ZnTe_80_Se. All structural relaxations were performed until the total energy converged to < 10^-5^ eV and the force on each atom was < 0.01 eV Å^-1^.

The defect formation energy (*E*_form_) was calculated by [6, 7]:

$E_{\text{form}}=E_{\text{defect}}-E_{\text{perfect}}+\sum_{i} n_{i}u_{i}$ (S1)

where *E*_defect_ and *E*_perfect_ are the total energies of defective and perfect supercells, respectively. *n*_i_ represents the number of added (*n*_i_ < 0) or removed (*n*_i_ > 0) atoms of type *i*; and*μ*_i_ is the corresponding chemical potential.

The calculation process for phonon dispersion and group velocity is as follows: First, structural optimization was performed on the pure phase Bi_2_Sb_10_Te_18_ and two types of Se-substituted structures (Bi_2_Sb_10_Te_17_Se, where Se replaces Te at specific sites) using first-principles calculations within the VASP [1]. The PBE generalized gradient approximation (GGA) functional was employed to treat exchange–correlation effects [5]. The plane-wave cutoff energy was set to 550 eV, with energy and force convergence criteria of 1×10^-8^ eV and 0.0001 eV/Å, respectively, to ensure accurate structural relaxation. Subsequently, based on the optimized crystal structures, the second-order force constant matrices were computed using phonoLAMMPS. In this step, a 5×5×5 supercell expansion was employed to accurately capture phonon interactions, and the interatomic potentials for Bi, Sb, and Te were selected from the nep89 potential set [8]. Finally, the Phonopy package was utilized to read the force constant matrices and generate the phonon dispersion spectra, thereby assessing the lattice dynamical stability of the structures [9].

**5. Construction and evaluation of the multifunctional TE device**

A full-scale TE device was fabricated using Bi_0.4_Sb_1.6_Te_2.97_Se_0.04_ + 0.15% ZnSb as the p-type legs, and n-type Bi_2_Te_2.7_Se_0.3_ (prepared by hot extrusion) as the n-type legs. The device comprised seven p–n pairs, each with a lateral dimension of 10 mm× 10 mm. To optimize device architecture, numerical simulations were conducted using COMSOL Multiphysics, a commercial finite element analysis (FEA) platform. The leg dimensions were set asymmetrically: p-type elements at 1.6 mm × 1.6 mm × 3 mm and n-type elements at 1.4 mm × 1.4 mm × 3 mm. Prior to assembly, all legs were coated with an electroplated Ni layer to serve as a diffusion barrier and were mounted in an alternating configuration onto a Cu-clad Al_2_O_3_ ceramic substrate. Electrical and thermal contacts were established using Sn_90_Sb_10_ solder (melting point ~260 °C).

The cooling performance of the TE device was evaluated by attaching it to a copper block equipped with a water circulation system using a thermally conductive adhesive. Hot-side (*T*_h_) and cold-side (*T*_c_) temperatures were monitored by K-type thermocouples. A ceramic heater (12 mm×12 mm), fixed atop the device with the same adhesive, allowed measurement of the cooling power (*Q*_c_) at various temperature differences (Δ*T* = *T*_h_ - *T*_c_). The minimum *T*_c_ was determined by adjusting the input current (*I*) to obtain the maximum cooling temperature difference (Δ*T*_max_). The coefficient of performance (COP) was calculated as COP = *Q*_c_/*P*_in_, where *P*_in_ is the total input power, and *Q*_cmax_ denotes the maximum cooling power achievable when Δ*T*_max_ = 0 K.

Power-generation characteristics were evaluated using a custom-designed measurement platform, calibrated against the Mini-PEM system (Advance Riko, Japan). For room-temperature output testing, one side of the device was placed on a heating platform while the other side was exposed to ambient air, with a thermal interface material applied to minimize thermal contact resistance. The ambient temperature was maintained at 293 K under draft-free conditions. Current-voltage (*I-V*) and output power (*P*) curves were recorded using a Keithley DMM6500 digital multimeter at heating-platform temperatures of 300 K, 304 K, 308 K, and 312 K.

**6. Effective mass modeling**

The electronic transport properties were modeled by treating the band structure as a single parabolic band (SPB) and acoustic phonon scattering as the dominant mechanism [10].

The *S* is expressed as:

$S=\frac{k_{B}}{e}[\frac{\left( r+5/2 \right)F_{r+3/2}\left( \eta\right)}{\left( r+3/2 \right)F_{r+1/2}\left( \eta\right)}-\eta]$ (S2)

where *η* is the reduced chemical potential, *k*_B_ is the Boltzmann constant, *e* is the elementary charge, and *r* = -1/2 is the scattering factor for acoustic phonons. The carrier concentration (*p*_H_) and Hall mobility ($\mu$_H_) are given by:

$p_{H}=\frac{16\pi\left( 2m_{d}^{*}k_{B}T \right)^{3/2}}{3h^{3}}\frac{F_{0}^{2}}{F_{-1/2}}$ (S3)

$\mu_{H}=\mu_{0}\frac{1}{2}\frac{F_{-1/2}}{F_{0}}$ (S4)

where $m_{d}^{*}$ is the density-of-states effective mass, determined from Pisarenco plots.

The *σ* can then be written as:

$\sigma=\frac{8\pi e\left( 2k_{B}T \right)^{3/2}m_{d}^{*3/2}\mu_{0}}{3h^{3}}F_{0}=\frac{8\pi e\left( 2k_{B}T \right)^{3/2}m_{e}^{-3/2}\mu_{w}}{3h^{3}}F_{0}$ (S5)

where $\mu_{w}=\left( m_{d}^{*}/m_{e} \right)^{3/2}\mu_{0}$.

The Lorentz factor (*L*) is given by:

$L=(\frac{k_{B}}{e})^{2}\frac{3F_{0}F_{2}-4F_{1}^{2}}{F_{0}^{2}}$ (S6)

with the Fermi integrals $F_{j}$ is defined by:

$F_{j}\left( \eta\right)=\int_{0}^{\infty} \frac{\xi^{j}d\xi}{1+e^{(\xi-\eta)}}$ (S7)

**7. Calculation of lattice thermal conductivity (***κ***_l_)**

The effective medium theory (EMT) is employed to correct the influence of medium losses on phonon transport. Microporous structures are treated as a special medium type, with the relationship between thermal conductivities of porous and dense samples expressed as [10-13]:

*κ*_p_ = *κ*_d_ *f_κ_*(*ε*) (S8)

where *κ*_p_ and *κ*_d_ denote the thermal conductivities of the porous and dense samples, respectively, and *ε* represents the void volume fraction. It has been reported that simplifying voids as spherical does not overestimate their effect on reducing the thermal conductivity. Thus, *f_κ_*(*ε*) can be expressed as:

*f_κ_*(*ε*) = 1-3*ε*/2 (S9)

The integrand term, along with the coefficient in Equation (S8), represents the spectral lattice thermal conductivity [14]:

$\text{κ}_{s}(\omega)=\frac{k_{B}}{2\pi^{2}\upsilon}\left( \frac{k_{B}T}{\hbar} \right)^{3}\tau_{tot}(x)\frac{x^{4}e^{x}}{{(e^{x}-1)}^{2}}$ (S10)

In the above equation, *x* = *ћω/k_B_T* is the reduced phonon frequency, $k_{B}$ is the Boltzmann constant, $\upsilon$ is the average sound velocity, calculated using $\upsilon={[\frac{1}{3}\left( \frac{1}{\upsilon_{L}^{3}}+\frac{1}{\upsilon_{T}^{3}} \right)]}^{\frac{-1}{3}}$, where $\upsilon_{L}$ and $\upsilon_{T}$ are the longitudinal and transverse sound velocities, respectively. $\hbar$ is reduced Plank’s constant, $\theta_{D}$ is the Debye temperature, and $\omega$ is the phonon frequency. The total relaxation time $\tau_{tot}$ is according to Matthiessen’s rule [15]:

$\tau_{tot}^{-1}=\tau_{UN}^{-1}+\tau_{PD}^{-1}+\tau_{GB}^{-1}+\tau_{SF}^{-1}+{}_{D}^{-1}$ (S11)

where *τ_UN_*, *τ_PD_*, *τ_GB_*, *τ_SF,_* and *τ_D_* represent the relaxation time of the Umklapp scattering, point defect scattering, grain boundary scattering, stacking fault scattering, and dislocation scattering, respectively. The relevant phonon relaxation time is given by:

Umklapp phonon-phonon scattering:

$\tau_{UN}^{-1}=A_{N}\frac{2}{\left( 6\pi^{2} \right)^{\frac{1}{3}}}\frac{k_{B}\bar{V}^{\frac{1}{3}}\gamma^{2}\omega^{2}T}{\bar{M}\upsilon^{3}}$ (S12)

Point defect phonon scattering:

$\tau_{PD}^{-1}=\frac{\bar{V}\omega^{4}}{4\pi\upsilon^{3}}\Gamma$ (S13)

Grain boundary phonon scattering:

$\tau_{GB}^{-1}=\frac{\upsilon}{d}$ (S14)

Here, *γ* is the Grüneisen parameter, $\bar{M}$ is the average mass, *m** is the effective mass of the charger carrier, *ρ* is the sample density, $\bar{V}$ is the average atomic volume, *Γ* is the point-defect scattering parameter (which is determined by considering only mass difference, a method known to work well for the Bi_2_Te_3_-Sb_2_Te_3_ system), and *d* is the grain size. The Umklapp phonon–phonon scattering strength coefficient *A*_N_ was fitted to experimental data of the in-plane *κ*_l_

Considering only specular phonon reflection at stacking faults, Klemens found that [16]:

$\tau_{SF}^{-1}=0.7\frac{a^{2}}{v}\gamma^{2}\omega^{2}N_{s}$ (S15)

where *a*, $v$, $\gamma$ and $N_{s}$ denote the lattice parameter, average sound speed, Grüneisen parameter, and the number of stacking faults per unit length, respectively.

Dislocation scattering includes both dislocation core (*τ*_DC_) and dislocation strain (*τ*_DS_) scattering:

${}_{D}^{-1}=\tau_{DC}^{-1}+\tau_{DS}^{-1}$ (S16)

${}_{DC}^{-1}=N_{D}\frac{\bar{V}^{4/3}}{\upsilon^{2}}{}^{3}$ (S17)

${}_{DS}^{-1}=0.6N_{D}{}^{2}{B_{D}}^{2}\left\{ \frac{1}{2}+\frac{1}{24}\left( \frac{1-2}{1-} \right)^{2}\left[ 1+\sqrt{2}\left( \frac{\upsilon_{L}}{\upsilon_{T}} \right)^{2} \right]^{2} \right\}$ (S18)

In these equations, *N_D_* is the number of dislocations crossing a unit length, and *B*_D_ is the magnitude of the Burgers vector of the dislocation. The detailed parameters are listed in Table S2.

**8. Calculation of the electronic quality factor (*B*_E_)**

For both *S* and *σ* at a fixed temperature, the electronic quality factor *B*_E_ can be defined [17]:

$B_{E}=S^{2}\sigma/\left[ \frac{S_{r}^{2}exp(2-S_{r})}{1+exp[-5\left( S_{r}-1 \right)]}+\frac{S_{r}\pi^{2}/3}{1+exp[5\left( S_{r}-1 \right)]} \right]$ (S19)

where $S_{r}=\frac{\left| S \right|}{k_{B}/e}$.

**9. 3D finite element model**

The main constitutive equations associated with the TE conversion in the TE coupling are as follows:

$J=-\sigma\alpha\nabla T-\sigma\nabla V$ (S20)

$q=T\alpha J-J\nabla V-\kappa\nabla T$ (S21)

Equation (S20) accounts for the contribution of the Seebeck effect to the current density. Equation (S21) describes the combined effects of the Seebeck, Peltier, and Joule heating effects on the heat flux.

Under steady-state conditions, the divergence of the current density is zero, and the corresponding boundary conditions can be written as:

$\nabla\cdot J=0$ (S22)

$\nabla\cdot q=-J\nabla V$ (S23)

Here, $J\nabla V$ represents the Joule heat flux generated by the circuit.

More explicitly, the TE equations are:

$\nabla\cdot(-\sigma\alpha\nabla T-\sigma\nabla V)=0$ (S24)

$\nabla\cdot(\alpha J-J\nabla V-\kappa\nabla T)=-(-\sigma\nabla V-\sigma\alpha\nabla T)\cdot(\nabla V)$ (S25)

**Supplementary Figures**


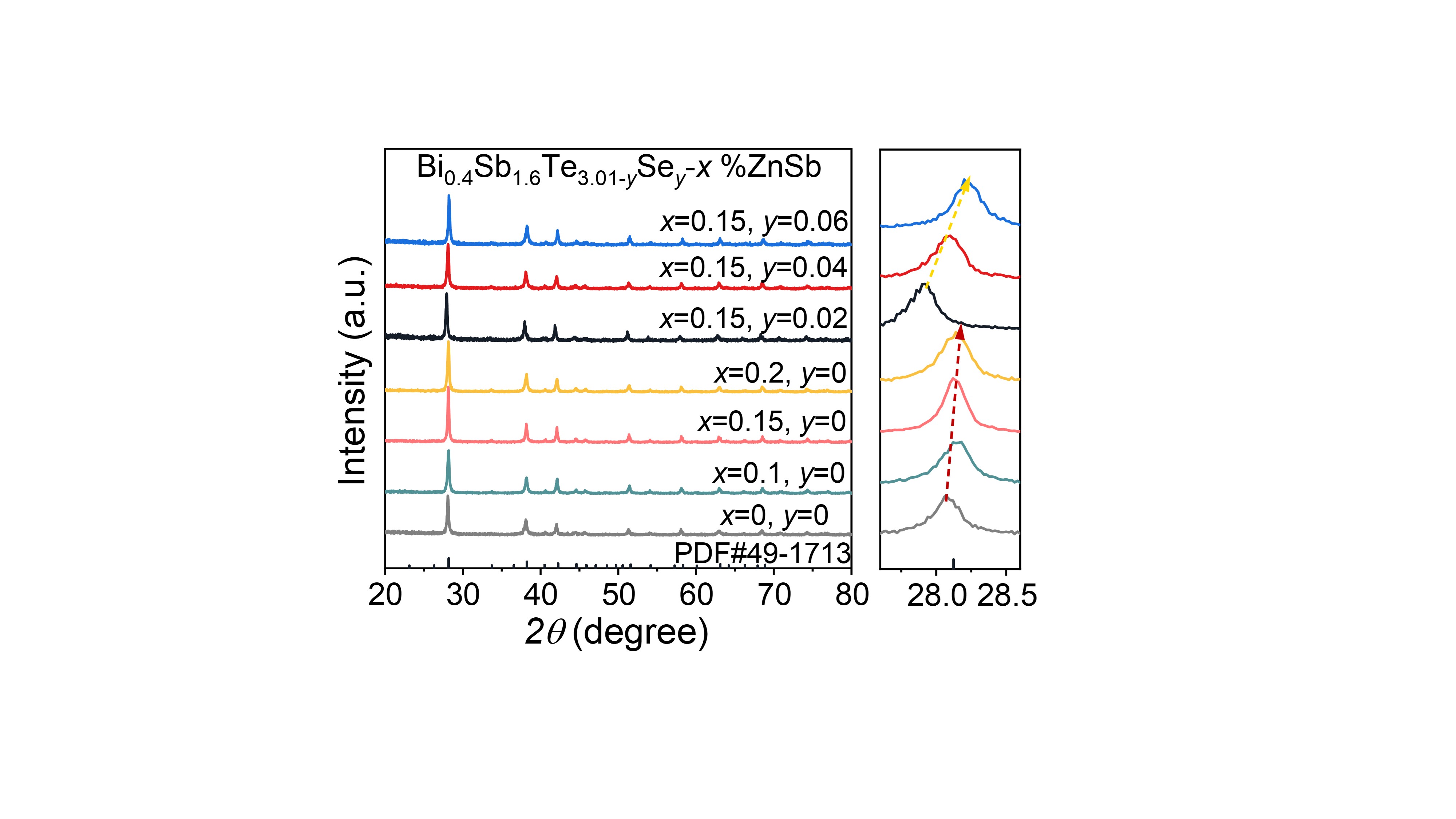


**Figure S1.** X-ray diffraction (XRD) patterns of Bi_0.4_Sb_1.6_Te_3.01-_*_y_*Se*_y_* + *x*% ZnSb samples with varying ZnSb contents (*x* = 0, 0.1, 0.15, 0.2) and Se doping levels (*y* = 0.02, 0.04, 0.06).


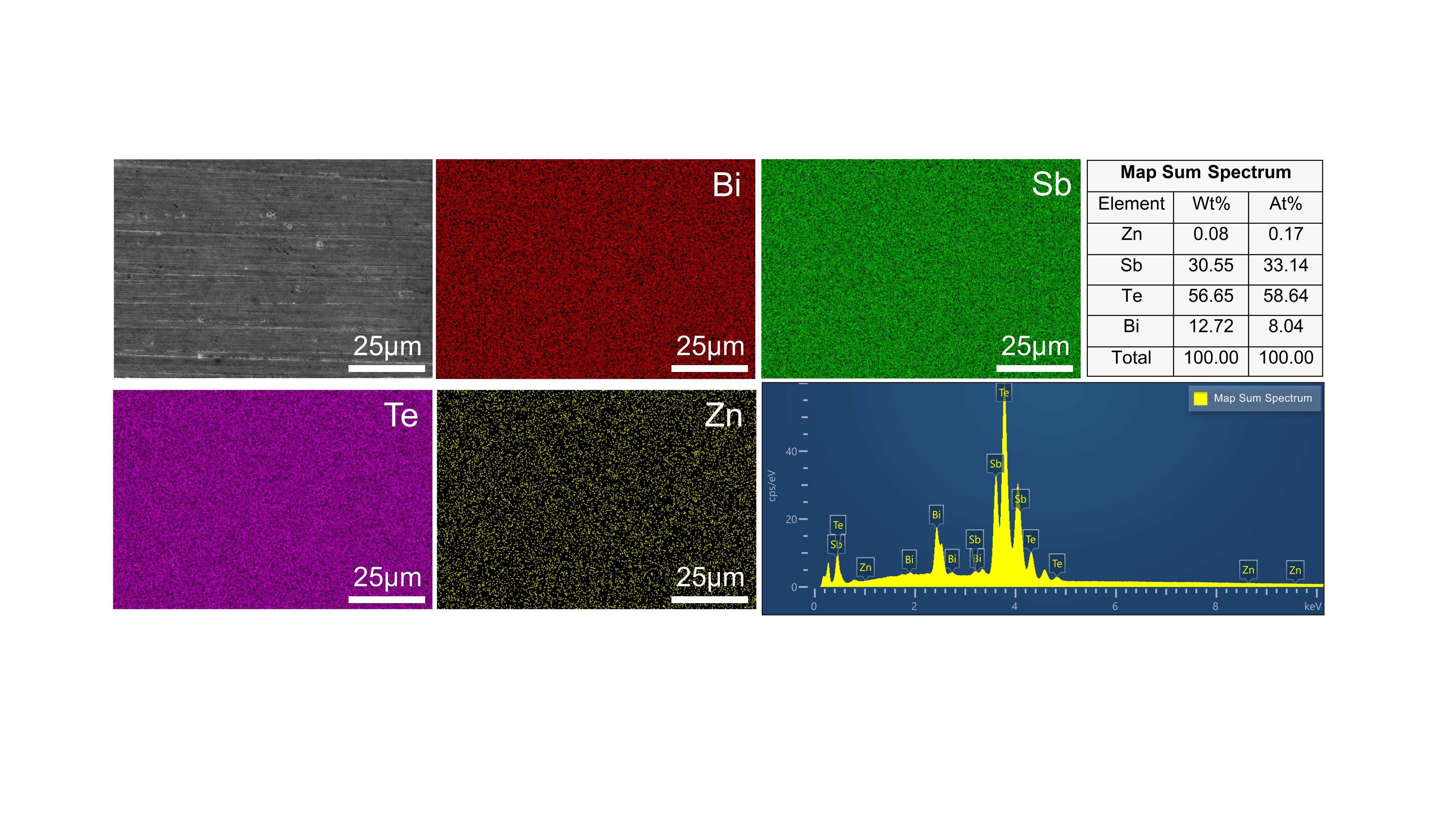


**Figure S2.** Back-scattered electron (BSE) image of the Bi_0.4_Sb_1.6_Te_3.01_ + 0.15% ZnSb sample together with the corresponding energy-dispersive X-ray spectroscopy (EDS) spectrum.


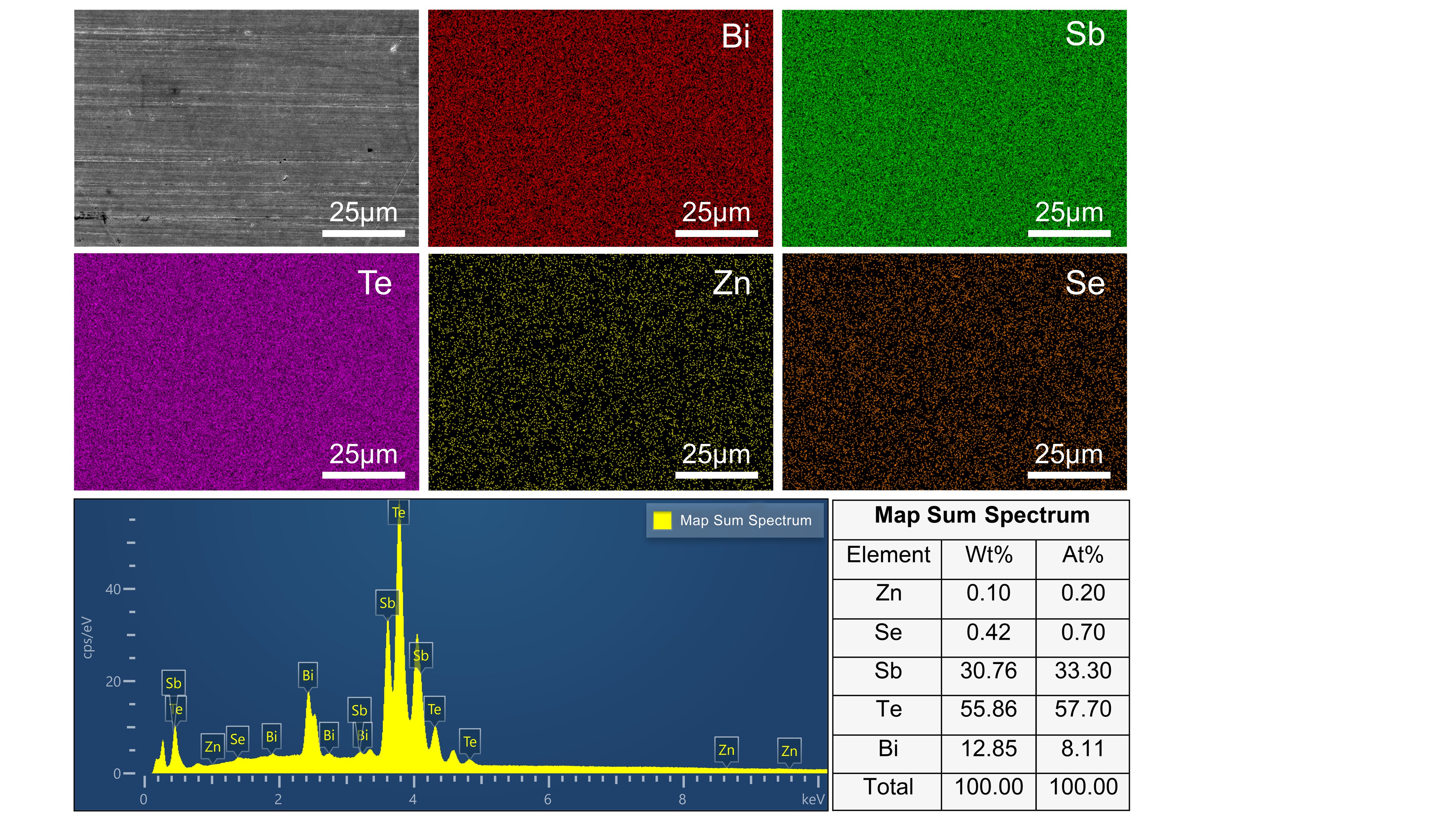


**Figure S3.** BSE image of the Bi_0.4_Sb_1.6_Te_2.97_Se_0.04_ + 0.15% ZnSb sample together with the corresponding EDS spectrum.


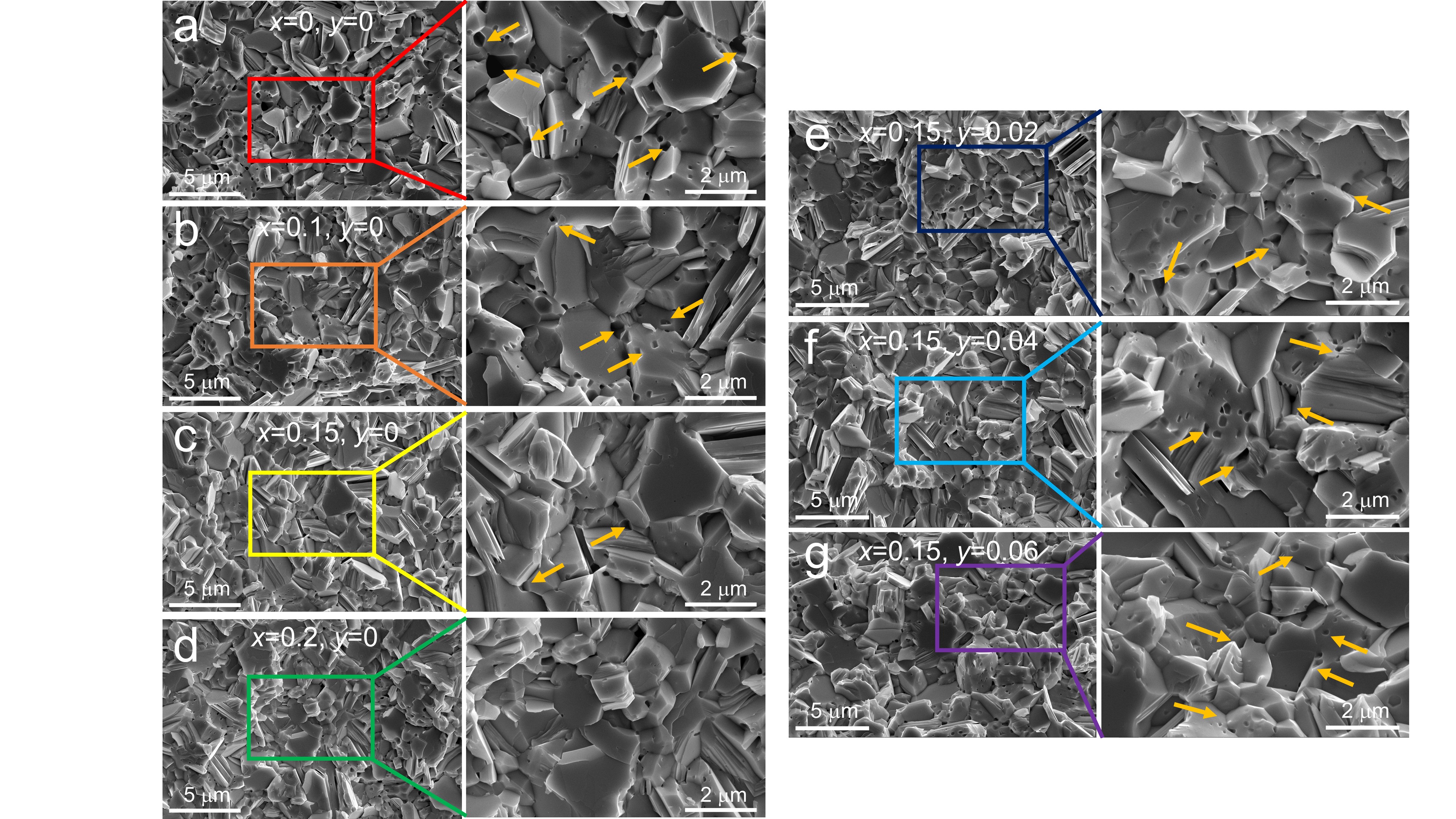


**Figure S4.** (a-g) Fracture surface images and corresponding magnified views of Bi_0.4_Sb_1.6_Te_3.01-_*_y_*Se*_y_* + *x*% ZnSb samples with varying ZnSb contents (*x* = 0, 0.1, 0.15, 0.2) and Se doping levels (*y* = 0.02, 0.04, 0.06).


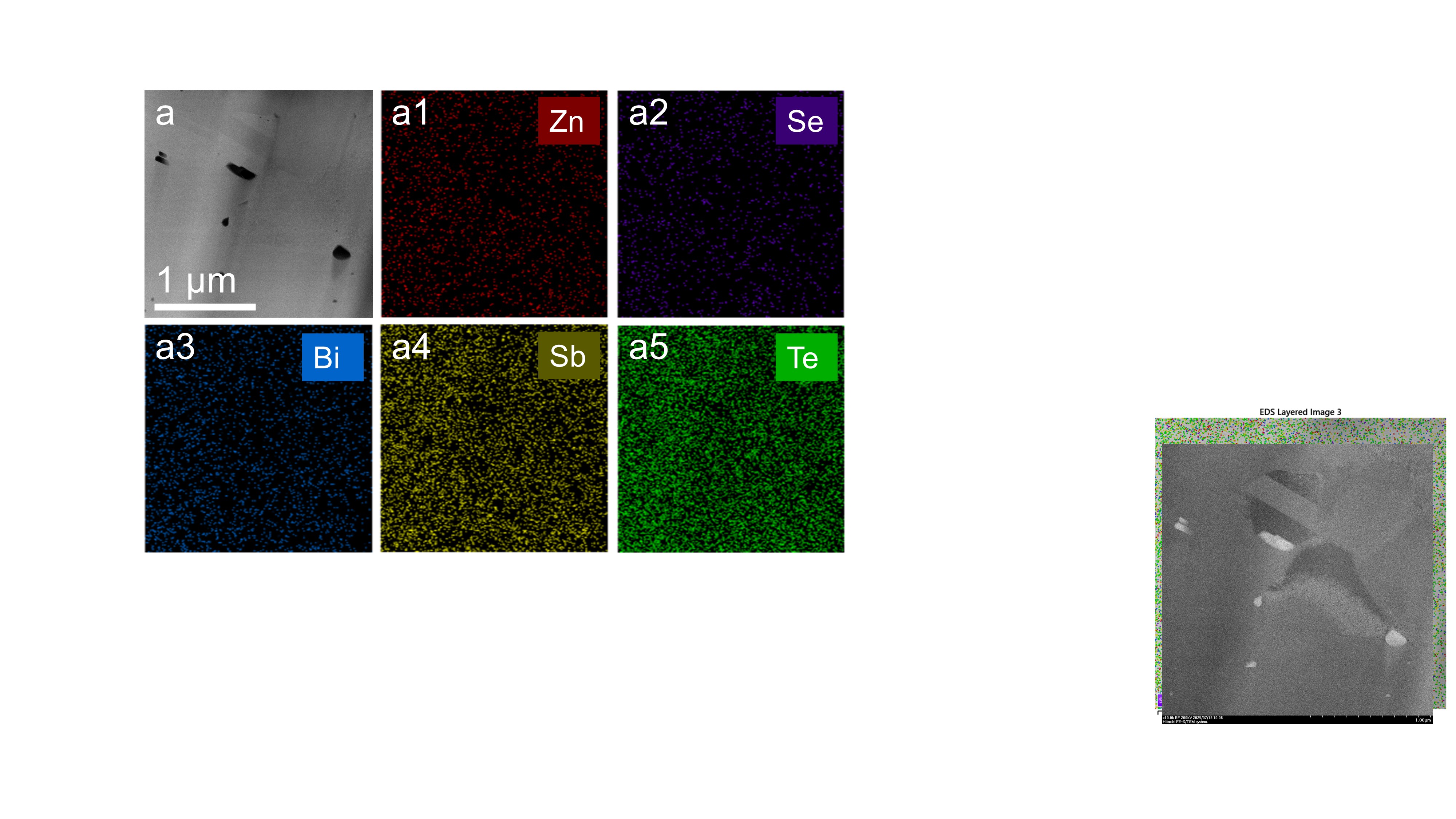

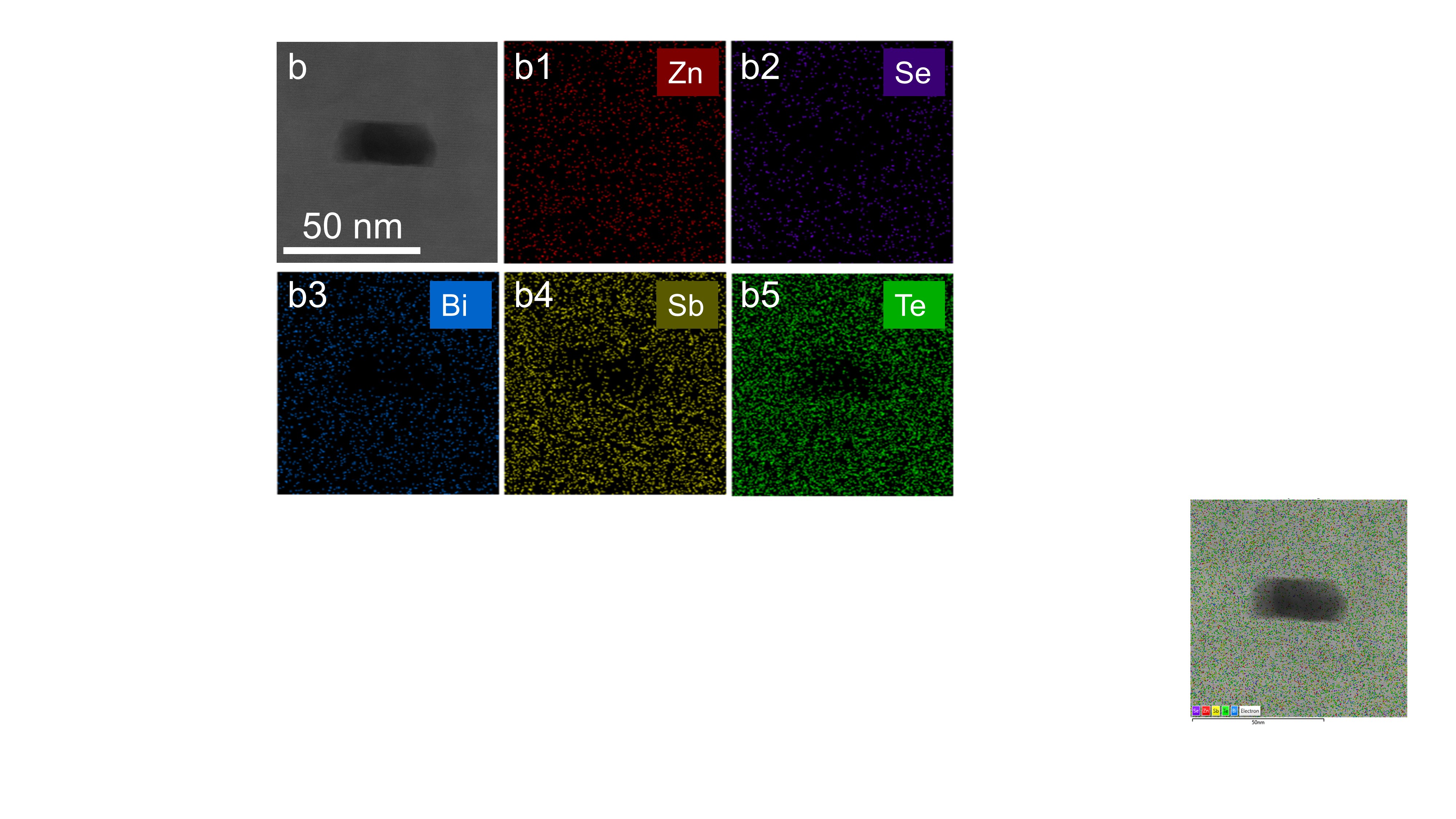


**Figure S5.** Micropores observed at different magnifications in the Bi_0.4_Sb_1.6_Te_2.97_Se_0.04_ + 0.15% ZnSb sample, together with the surrounding scanning transmission electron microscopy (STEM) images and the corresponding EDS elemental maps.


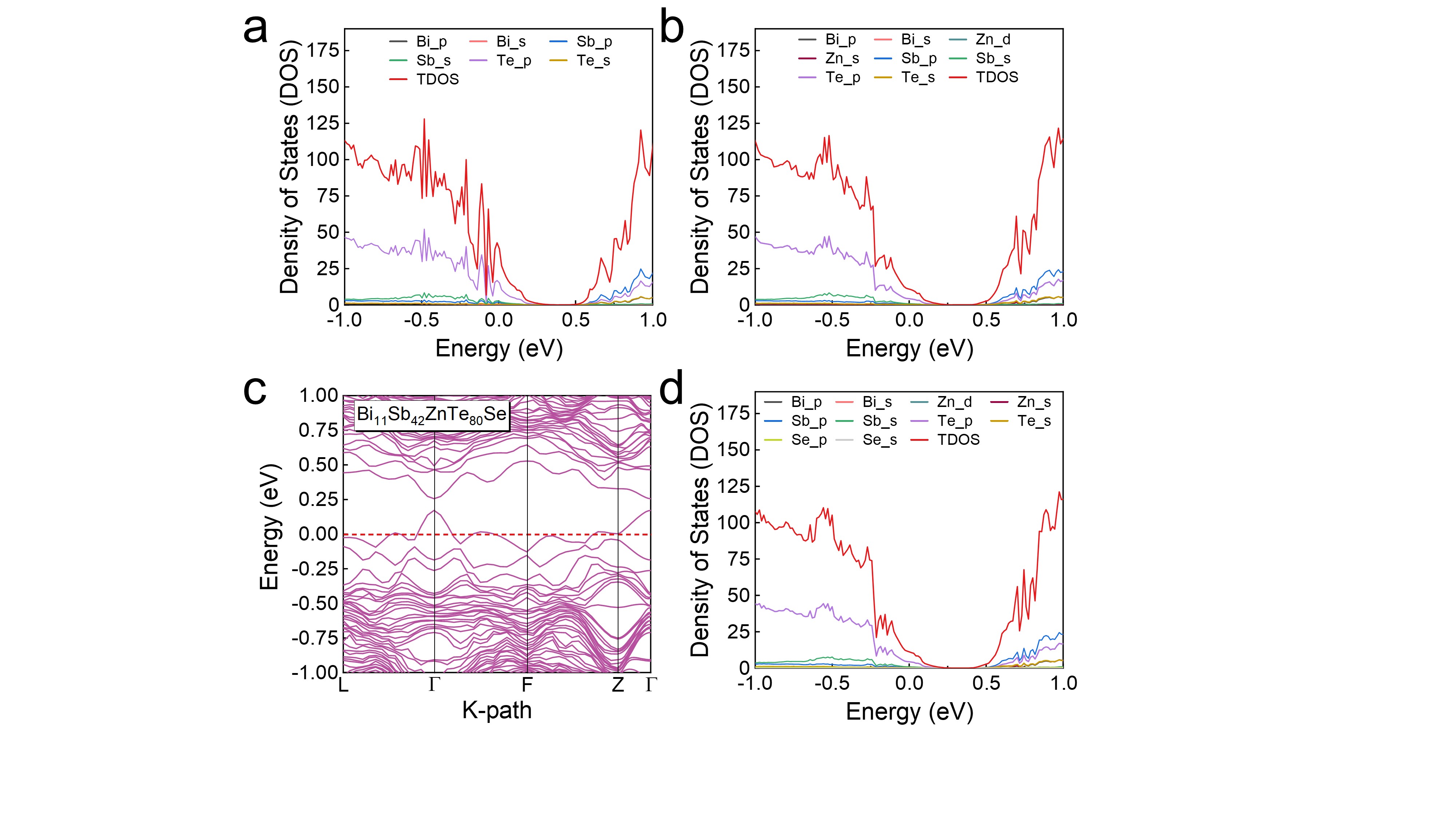


**Figure S6.** Calculated electronic density of states (DOS) for (a) Bi_11_Sb_42_Te_81_, (b) Bi_11_Sb_42_ZnTe_81_, and (d) Bi_11_Sb_42_ZnTe_80_Se. (c) Calculated electronic band structure for Bi_11_Sb_42_ZnTe_80_Se.


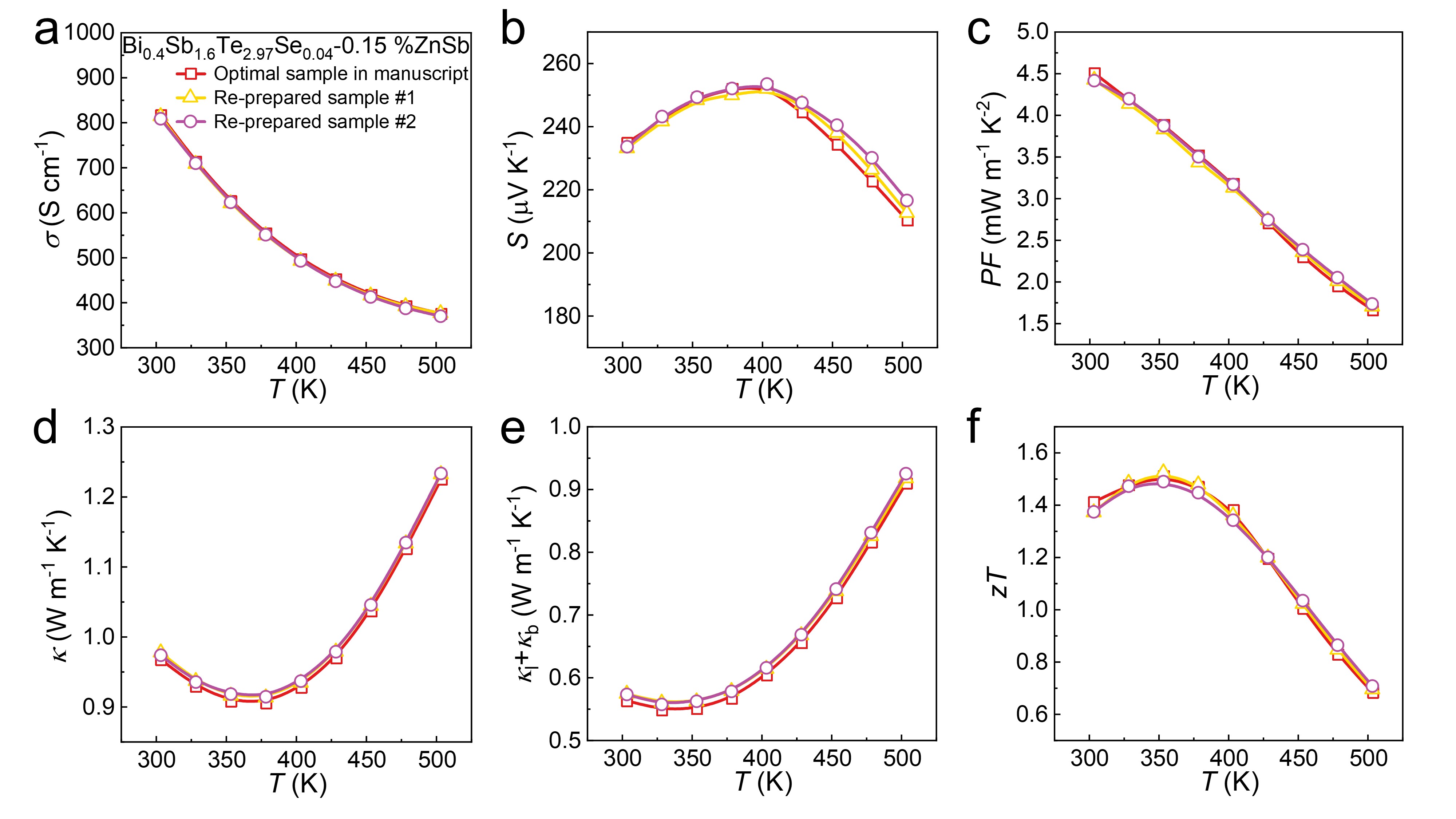


**Figure S7.** Transport properties of the re-prepared samples. Temperature dependence of (a) electrical conductivity (*σ*), (b) Seebeck coefficient (*S*), (c) power factor (*PF*), (d) total thermal conductivity (*κ*), (e) lattice and bipolar thermal conductivity (*κ*_l_ + *κ*_b_), and (f) the dimensionless figure of merit (*zT*).


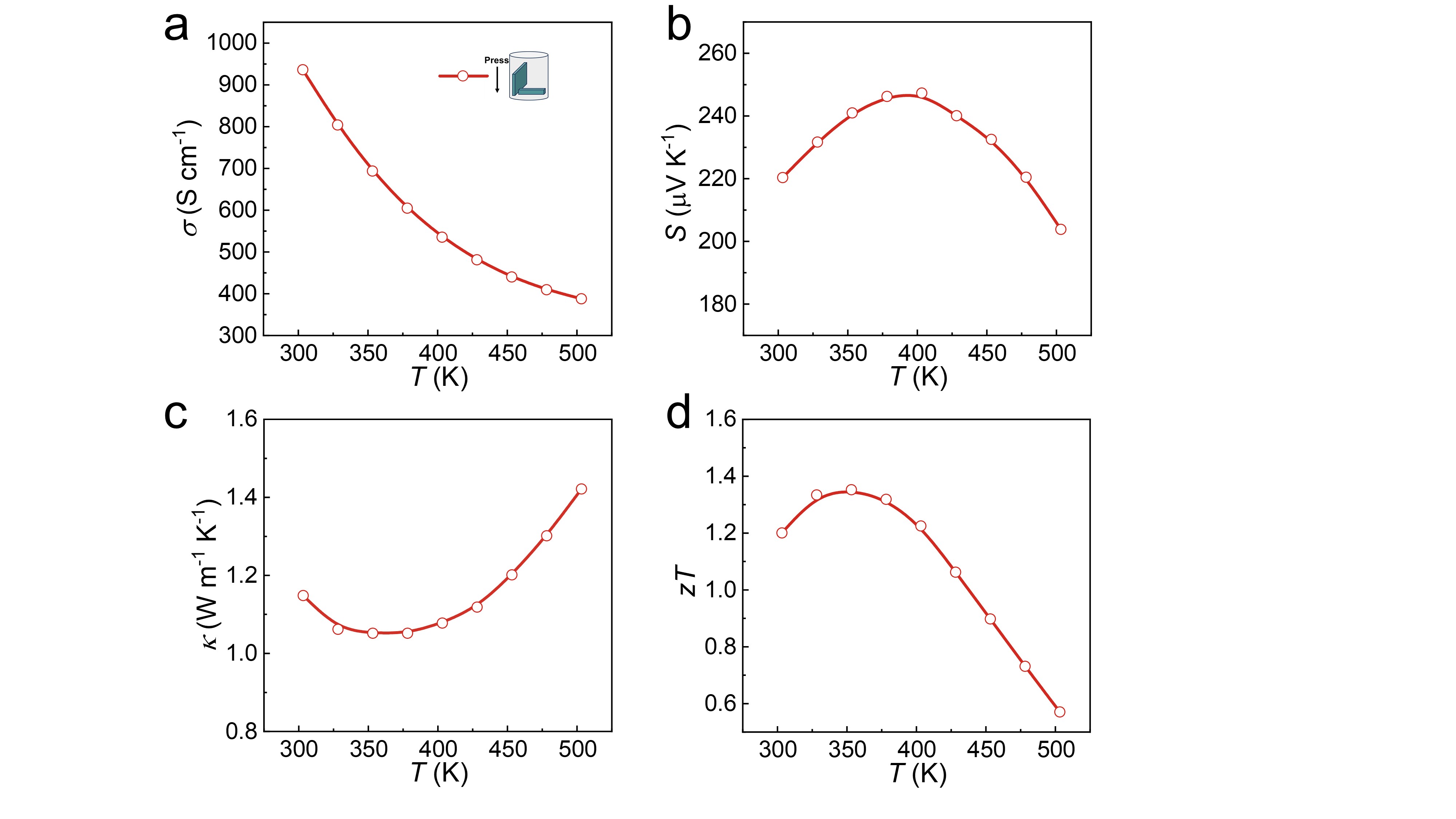


**Figure S8.** TE properties of Bi_0.4_Sb_1.6_Te_2.97_Se_0.04_ + 0.15% ZnSb sample measured perpendicular to the hot-pressing direction: (a) *σ*, (b) *S*, (c) *κ*, and (d) *zT*.


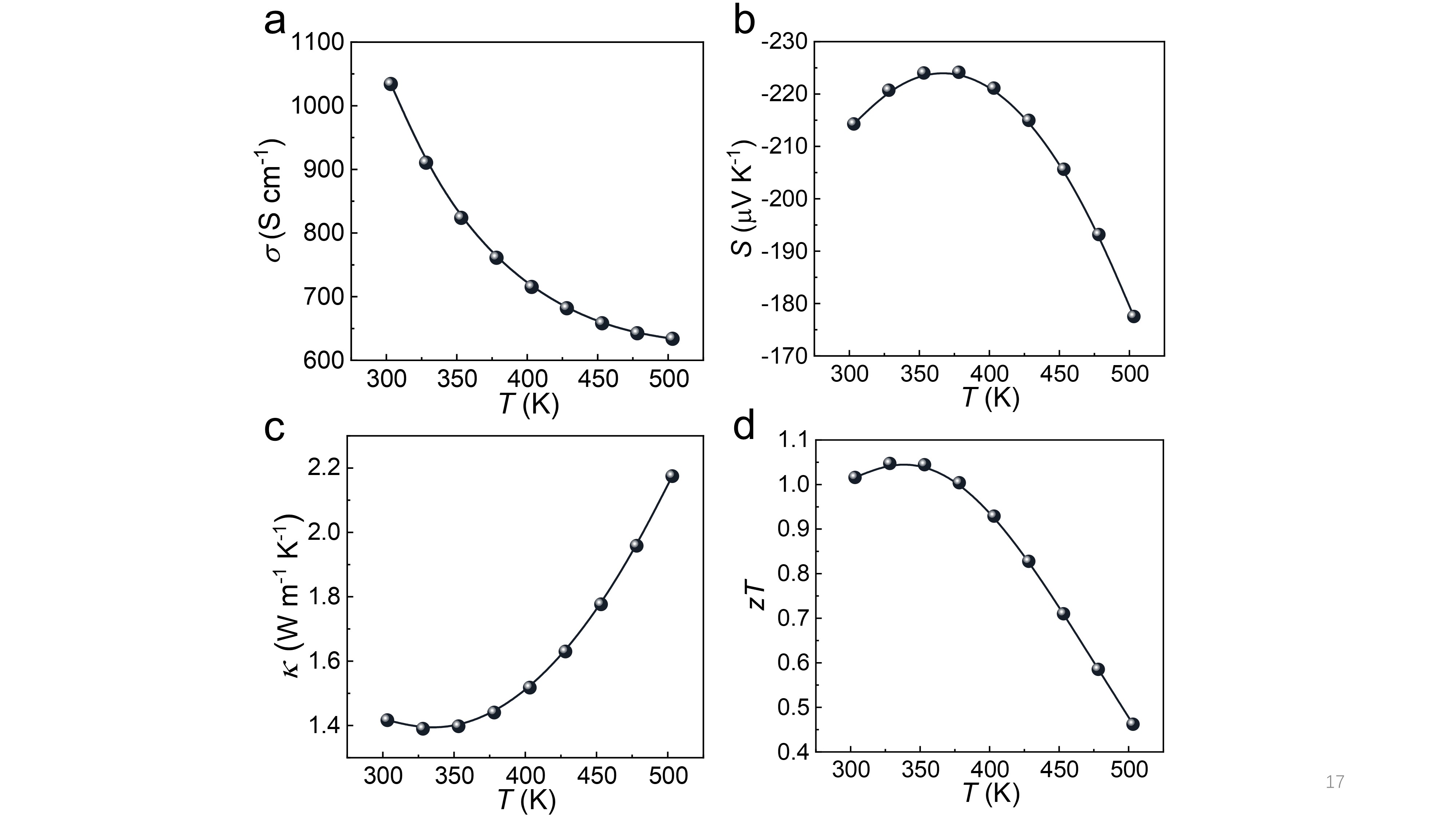


**Figure S9.** Temperature-dependent TE properties of *n*-type Bi_2_Te_2.7_Se_0.3_ sample: (a) *σ*, (b) *S*, (c) *κ*, and (d) *zT*.


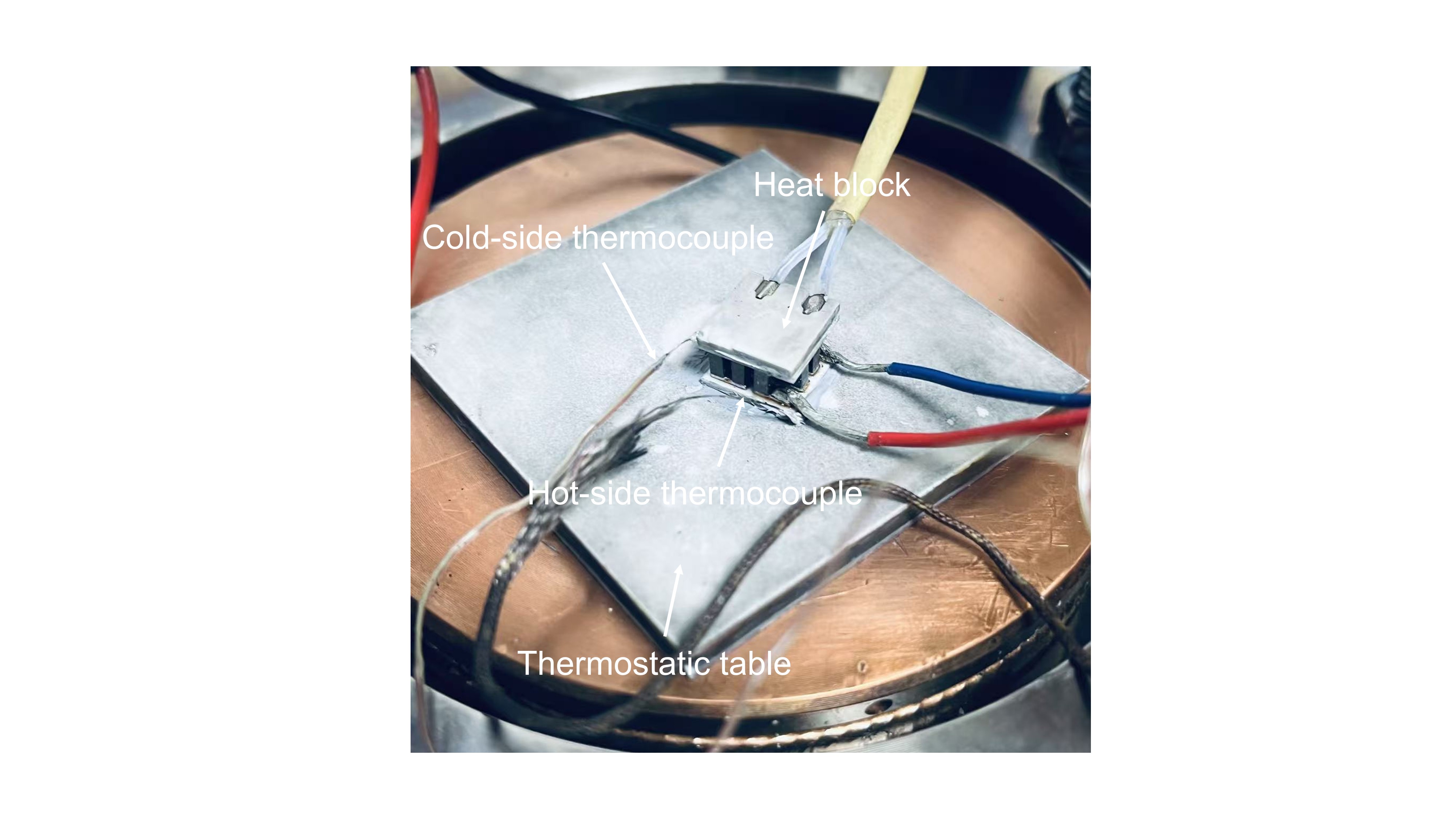


**Figure S10.** Photograph of the TE cooling test setup.


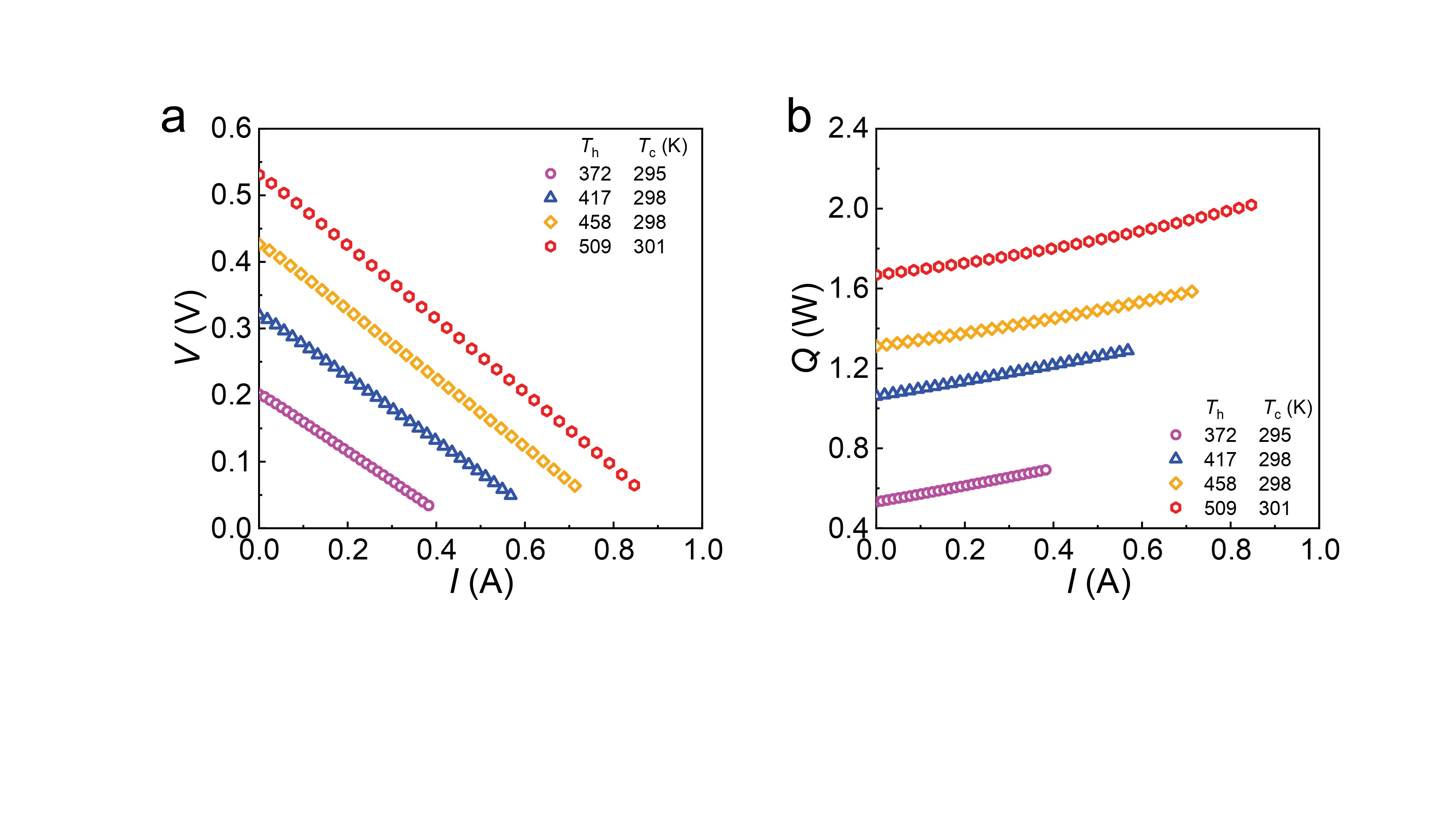


**Figure S11.** (a) voltage (*V*) and (b) heat flow (*Q*) as a function of current (*I*) at different *T*_h_ and *T*_c_ for the seven-pair TE devices.

**Table S1.** Density of Bi_0.4_Sb_1.6_Te_3.01-_*_y_*Se*_y_* + *x*% ZnSb samples prepared in this work, with varying ZnSb contents (*x* = 0, 0.1, 0.15, 0.2) and Se doping levels (*y* = 0.02, 0.04, 0.06).

| Composition | Measured density  (g cm^-3^) | Relative density  (%) |
| --- | --- | --- |
| Bi_0.4_Sb_1.6_Te_3.01_ | 6.611 | 97.5 |
| Bi_0.4_Sb_1.6_Te_3.01_ + 0.1 %ZnSb | 6.594 | 97.2 |
| Bi_0.4_Sb_1.6_Te_3.01_ + 0.15 %ZnSb | 6.591 | 97.2 |
| Bi_0.4_Sb_1.6_Te_3.01_ + 0.2 %ZnSb | 6.584 | 97.1 |
| Bi_0.4_Sb_1.6_Te_2.99_Se_0.02_ + 0.15 %ZnSb  Bi_0.4_Sb_1.6_Te_2.97_Se_0.04_ + 0.15 %ZnSb  Bi_0.4_Sb_1.6_Te_2.95_Se_0.06_ + 0.15 %ZnSb | 6.578  6.567  6.557 | 97.0  96.9  96.7 |

**Table S2.** Parameters used for the calculation of lattice thermal conductivity (*κ*_l_).

| Parameters | Description | Values | | Ref. |
| --- | --- | --- | --- | --- |
| $\theta_{D}$ | Debye temperature | 124 | | Ref.[18] |
| $v$ | Average sound velocity | 1922 m·s^-1^ | | Ref.[19] |
| $A_{N}$ | Comprehensive coefficient between  Umklapp and Normal processes | 2.3 | | fitted |
| $\bar{V}$ | Average atomic volume of Bi_0.4_Sb_1.6_Te_3_ | 3.23×10^-29^ m^3^ | | Ref.[20] |
| $\bar{M}$ | Average atomic mass of Bi_0.4_Sb_1.6_Te_3_ | 2.2×10^-25^ kg | | fitted |
| $\gamma$ | Grüneisen parameter | 1.47 | | Ref.[19] |
| $r$ | Poisson's ratio | 0.25 | | Ref.[18] |
| $v_{L}$ | Longitudinal sound velocity | 2979 m·s^-1^ | | Ref.[19] |
| $v_{T}$ | Transverse sound velocity | 1733 m·s^-1^ | | Ref.[19] |
| $\Gamma$ | Point defect scattering parameter | 0.175 | | Ref.[18] |
| d | Grain size | 3.2×10^-6^ m | | Exp. |
| *N*_s_ | Number of stacking faults | | 8.9×10^6^ m^-1^ | Exp. |
| *N*_D_ | Dislocation density | 6.3×10^10^ cm^-2^ | | Exp. |
| *B*_D_ | Magnitude of Burger's vector | 12.7 Å | | Ref.[18] |

**References**

1. G. Kresse, J. Furthmüller, “Efficient Iterative Schemes for Ab Initio Total-Energy Calculations Using a Plane-Wave Basis Set,” *Physical Review B* 54 (1996): 11169, <https://doi.org/10.1103/PhysRevB.54.11169>.

2. J. Furthmüller, J. Hafner, G. Kresse, “Dimer Reconstruction and Electronic Surface States on Clean and Hydrogenated Diamond (100) Surfaces.,” *Physical Review B* 53 (1996): 7334, <https://doi.org/10.1103/PhysRevB.53.7334>.

3. G. Kresse, J. Furthmüller, “Efficiency of Ab-Initio Total Energy Calculations for Metals and Semiconductors Using a Plane-Wave Basis Set,” *Computational Materials Science* 6 (1996): 15, <https://doi.org/10.1016/0927-0256(96)00008-0>.

4. V. Wang, N. Xu, J.-C. Liu, G. Tang, W.-T. Geng, “VASPKIT: A User-Friendly Interface Facilitating High-Throughput Computing and Analysis Using VASP Code,” *Computer Physics Communications* 267 (2021): 108033, <https://doi.org/10.1016/j.cpc.2021.108033>.

5. J. P. Perdew, K. Burke, M. Ernzerhof, “Generalized Gradient Approximation Made Simple,” *Physical Review Letters* 77 (1996): 3865, <https://doi.org/10.1103/PhysRevLett.77.3865>.

6. C. G. Van de Walle, J. Neugebauer, “First-Principles Calculations for Defects and Impurities: Applications to III-Nitrides,” *Journal of Applied Physics* 95 (2004): 3851, <https://doi.org/10.1063/1.1682673>.

7. S. B. Zhang, “The Microscopic Origin of The Doping Limits in Semiconductors and Wide-Gap Materials and Recent Developments in Overcoming These Limits: A Review,” *Journal of Physics: Condensed Matter* 14 (2002): R881, <https://doi.org/10.1088/0953-8984/14/34/201>.

8. T. Liang, K. Xu, E. Lindgren, et al., “NEP89: Universal Neuroevolution Potential for Inorganic and Organic Materials Across 89 Elements,” *arXiv* 2504: 21286, <https://doi.org/10.48550/arXiv.2504.21286>

9. A. Togo, L. Chaput, T. Tadano, I. Tanaka, “Implementation Strategies in Phonopy and Phono3py,” *Journal of Physics: Condensed Matter* 35 (2023): 353001, <https://doi.org/10.1088/1361-648X/acd831>.

10. Y. Pan, U. Aydemir, J. A. Grovogui, et al., “Melt‐Centrifuged (Bi,Sb)_2_Te_3_: Engineering Microstructure toward High Thermoelectric Efficiency,” *Advanced Materials* 30 (2018): 1802016 <https://doi.org/10.1002/adma.201802016>.

11. D. Stroud, “Generalized Effective-Medium Approach to the Conductivity of an Inhomogeneous Material,” *Physical Review B* 12 (1975): 3368, <https://doi.org/10.1103/PhysRevB.12.3368>.

12. C.-W. Nan, R. Birringer, D. R. Clarke, H. Gleiter, “Effective Thermal Conductivity of Particulate Composites with Interfacial Thermal Resistance,” *Journal of Applied Physics* 81 (1997): 6692, <https://doi.org/10.1063/1.365209>.

13. H. J. Juretschke, R. Landauer, J. A. Swanson, “Hall Effect and Conductivity in Porous Media,” *Journal of Applied Physics* 27 (1956): 838, <https://doi.org/10.1063/1.1722496>.

14. S. I. Kim, K. H. Lee, H. A. Mun, et al., “Dense Dislocation Arrays Embedded in Grain Boundaries for High-Performance Bulk Thermoelectrics,” *Science* 348 (2015): 109, <https://doi.org/10.1126/science.aaa4166>.

15. T. Zhu, C. Fu, H. Xie, et al., “Lattice thermal conductivity and spectral phonon scattering in FeVSb-based half-Heusler compounds,” *EPL (Europhysics Letters)* 104 (2013): 46003, [https://doi.org/10.1209/0295-5075/104/46003](https://doi.org/10.1039/d2ee00119e).

16. B. K. Singh, V. J. Menon, K. C. Sood, “Phonon Conductivity of Plastically Deformed Crystals: Role of Stacking Faults and Dislocations,” *Physical Review B* 74 (2006): 184302, <https://doi.org/10.1103/PhysRevB.74.184302>.

17. X. Zhang, Z. Bu, X. Shi, et al., “Electronic Quality Factor for Thermoelectrics,” *Science Advances* 6 (2020): eabc0726, <https://doi.org/10.1126/sciadv.abc0726>.

18. H.-L. Zhuang, H. Hu, J. Pei, et al., “High ZT in p-Type Thermoelectric (Bi,Sb)_2_Te_3_ with Built-in Nanopores,” *Energy & Environmental Science* 15 (2022): 2039, <https://doi.org/10.1039/d2ee00119e>.

19. R. Li, X. L. Shi, J. Zhu, et al., “Cu_3_SbSe_3_‐Alloying‐Induced High Thermoelectric Performance and Mechanical Robustness in Bi_2_Te_3_‐Based Thermoelectric Materials,” *Advanced Science* 12 (2025): e12417, <https://doi.org/10.1002/advs.202512417>.

20. Q. Zhang, M. Yuan, K. Pang, et al., “High‐Performance Industrial‐Grade p‐Type (Bi,Sb)_2_Te_3_ Thermoelectric Enabled by a Stepwise Optimization Strategy,” *Advanced Materials* 35 (2023): 2300338, <https://doi.org/10.1002/adma.202300338>.
